# *Drosophila* Imp iCLIP identifies an RNA assemblage coordinating F-actin formation

**DOI:** 10.1186/s13059-015-0687-0

**Published:** 2015-06-09

**Authors:** Heidi Theil Hansen, Simon Horskjær Rasmussen, Sidsel Kramshøj Adolph, Mireya Plass, Anders Krogh, Jeremy Sanford, Finn Cilius Nielsen, Jan Christiansen

**Affiliations:** Department of Biology, Center for Computational and Applied Transcriptomics, University of Copenhagen, Ole Maaloes Vej 5, 2200 Copenhagen, Denmark; MCD Biology, University of California, Santa Cruz, CA 95064 USA; Center for Genomic Medicine, Rigshospitalet, University of Copenhagen, 2100 Copenhagen, Denmark

## Abstract

**Background:**

Post-transcriptional RNA regulons ensure coordinated expression of monocistronic mRNAs encoding functionally related proteins. In this study, we employ a combination of RIP-seq and short- and long-wave individual-nucleotide resolution crosslinking and immunoprecipitation (iCLIP) technologies in *Drosophila* cells to identify transcripts associated with cytoplasmic ribonucleoproteins (RNPs) containing the RNA-binding protein Imp.

**Results:**

We find extensive binding of Imp to 3′ UTRs of transcripts that are involved in F-actin formation. A common denominator of the RNA–protein interface is the presence of multiple motifs with a central UA-rich element flanked by CA-rich elements. Experiments in single cells and intact flies reveal compromised actin cytoskeletal dynamics associated with low Imp levels. The former shows reduced F-actin formation and the latter exhibits abnormal neuronal patterning. This demonstrates a physiological significance of the defined RNA regulon.

**Conclusions:**

Our data imply that *Drosophila* Imp RNPs may function as cytoplasmic mRNA assemblages that encode proteins which participate in actin cytoskeletal remodeling. Thus, they may facilitate coordinated protein expression in sub-cytoplasmic locations such as growth cones.

**Electronic supplementary material:**

The online version of this article (doi:10.1186/s13059-015-0687-0) contains supplementary material, which is available to authorized users.

## Background

To establish a neuronal network, protrusions, in the form of filopodia and lamellipodia, project from the developing axons into their surroundings in search of their appropriate postsynaptic targets. These protrusions are formed at the edge of the outermost part of the axon, the growth cone. This highly motile domain, consisting of bundles of filamentous actin, microtubules and their associated proteins, is an exquisite sensor of chemical gradients and is constantly undergoing dynamic structural changes in response to the surrounding guidance cues (reviewed in [1]). As the continuous remodeling of the growth cone requires the localization of proteins to the tip, synchronous on-site translation of mRNAs makes it possible to swiftly produce a subset of proteins needed for accurate responses to the guidance cues (reviewed in [2]).

**Fig. 1:**
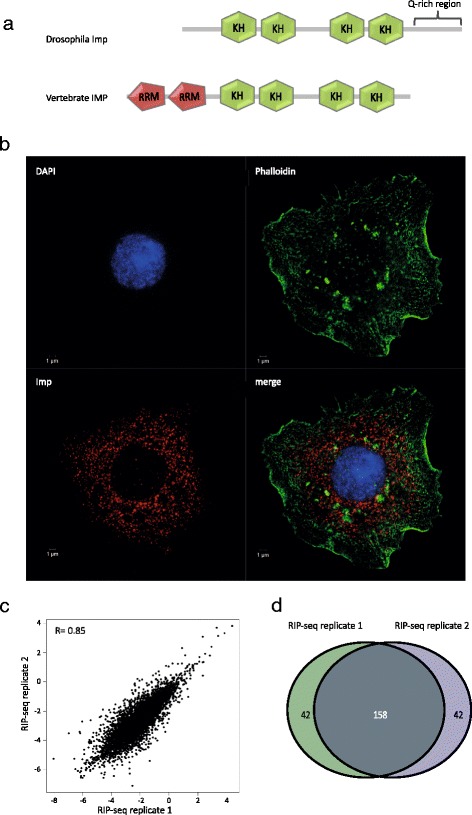
*Drosophila* Imp is present in cytoplasmic RNPs. **a** Protein domain architecture of *Drosophila* Imp and homologous vertebrate IMPs. The four KH domains are conserved whereas the glutamine (Q)-rich region and the two RNA recognition motifs (*RRM*) are structural features present in arthropods and chordates, respectively. **b** Immunocytochemistry of S2 cell with DAPI staining (*blue*), anti-Imp antibody (*red*) and phalloidin (*green*). **c** Correlation between the enrichment of immunoprecipitated transcripts in Imp RNPs from two biological RNA-binding protein immunoprecipitation (RIP)-seq replicates. The scatter plot shows the natural logarithm of RIP enrichment values per nucleotide in the whole transcript for each RIP-seq replicate. **d** Venn diagram of the 200 most enriched transcripts in Imp RNPs from two biological RIP-seq replicates

The post-transcriptional RNA regulon/operon hypothesis posits that functionally related monocistronic mRNAs are co-regulated by common *trans*-acting RNA-binding proteins (RBPs) [3]. The distinct binding profiles of yeast RNA-binding Puf isomers provide a striking example of how this type of regulation works with regard to common function and mRNA localization [4]. In situ hybridization studies in *Drosophila* blastoderms have estimated that 71 % of 3370 examined mRNAs are localized in diverse patterns [5], so the interplay between RBPs and target mRNAs is pervasive. However, most localization studies have focused on a single mRNA species and a corresponding RBP, exemplified by the β-actin mRNA/ZBP1 paradigm in fibroblasts and growth cones [6, 7]. ZBP1 belongs to a vertebrate family of RNA-binding proteins (other acronyms include IGF2BP, VICKZ, IMP) with a domain architecture of two RNA recognition motifs and four K homology (KH) domains, the latter providing the RNA attachment platform [8, 9]. The majority of vertebrate genomes contain three paralogs of this particular RBP family, whereas a single locus on the X chromosome encodes the *Drosophila* homolog Imp (insulin-like growth factor II mRNA-binding protein), containing the four conserved KH domains, but lacking the N-terminal two RNA recognition motifs. In addition, the *Drosophila* homolog encompasses a C-terminal Gln-rich extension that is not observed among vertebrates (Fig. 1a). Imp is expressed in the oocyte, the blastoderm embryo as well as in the developing nervous system and pole cells [10–12]. During mid-oogenesis, maternal Imp co-localizes with both *gurken* and *oskar* mRNAs, which are critical for dorsoventral and anteriorposterior axis formation, respectively [11, 13]. However, the functional significance of Imp in axis formation is uncertain since localization of both *gurken* and *oskar* mRNAs is unaffected in an Imp-deficient background, whereas Imp overexpression disrupts the localization and translational regulation of both transcripts. At later stages of development, increased pharate adult lethality is observed in Imp mutants, which is a phenotype often associated with defective synaptic transmission. More specifically, Imp mutant larvae exhibited smaller synaptic junctions [14], and a recent study revealed that extensive remodeling of γ-neurons in the mushroom body during pupation is jeopardized in the absence of Imp. It is not the initial growth of the axons that is compromised but their remodeling in terms of both length and directionality, and overexpression of the profilin homolog Chickadee is able to partially rescue the remodeling defect [15]. Imp is also expressed in the tail end of cyst cells surrounding elongating spermatids, and both spermatids and cyst cells elongate to an extreme length in an F-actin dependent fashion [12].

In recent years, high-throughput cross-linking and immunoprecipitation (CLIP) procedures have been introduced to obtain information regarding direct binding sites for individual RBPs on a transcriptome-wide scale in vivo [16–18]. In this way, novel details of DGCR8 (DiGeorge syndrome critical region 8) functions [19], regulation of splicing [16, 20], and alternative polyadenylation [21] have been elucidated. The salient feature of these procedures is the ability to identify the exact cross-linked position on a global scale by using stringent washing regimens to minimize non-covalent associations.

In the present study, we employed three different high-throughput procedures to identify the full targetome of *Drosophila* Imp and thus assess the post-transcriptional RNA regulon hypothesis. The data revealed extensive 3′ untranslated region (UTR) attachments in mRNAs encoding components crucial for actin cytoskeletal remodeling, and subsequent Imp knock-down analysis showed decreased F-actin levels in S2 cells. Moreover, an Imp-deficient fly exhibited a phenotype with difficulties reaching the pharate adult stage, mainly due to defects in the peripheral nervous system. Taken together, our study identifies an Imp-mediated RNA assemblage containing transcripts that encode proteins coordinating F-actin formation.

## Results

### *Drosophila* Imp localizes to cytoplasmic granules containing multiple mRNA species

To address the subcellular localization and potential of forming RNP granules in the *Drosophila* S2 cell line, immunostaining of endogenous Imp was performed with co-staining with phalloidin (Fig. 1b). Immunostaining revealed punctate fluorescence with an optical diameter of about 200 nm dispersed throughout the cytoplasm with limited nuclear Imp immunoreactivity, suggesting Imp localization in predominantly cytoplasmic RNP granules.

To characterize the repertoire of RNA targets that are associated with the Imp granules in *Drosophila* S2 cell lysates, we sequenced Imp-associated mRNAs following immunoprecipitation from cytoplasmic extracts using anti-Imp antibody-coated Protein A Dynabeads. Mapping statistics are listed in Figure S1a in Additional file 1. The enrichment factor (the ratio of immunoprecipitated RNA:input RNA) for each transcript was calculated, and the correlation of the values between the biological replicates was plotted. As shown in Fig. 1c, the replicate experiments are strongly correlated (R = 0.85), underscoring the precision of our measurements. Of the top 200 ranked transcripts in each biological replicate, 158 were present in both replicates (Fig. 1d). Gene ontology (GO) term analysis was performed on the 158 transcripts and showed enrichment in GO terms involved in neuronal development, reproduction, and cytoskeletal organization (Figure S1b in Additional file 1). This result is in agreement with earlier observations of Imp being implicated in ageing in the testis stem-cell niche [22], in spermatid cyst cell elongation [12], and in axonal remodeling in mushroom body neurons [15].

### iCLIP and PAR-iCLIP identify direct Imp target sites in 3′ UTRs

Since RIP-seq identifies both direct and indirect RNA targets without information regarding the position of interaction, we performed individual-nucleotide resolution cross-linking (iCLIP) [23] as well as a modified version of photoactivatable ribonucleoside-CLIP (PAR-CLIP) [17], which we have called PAR-iCLIP, on cytoplasmic extracts of S2 cells to pinpoint the positions of direct binding. We generated two biological replicate iCLIP and two biological replicate PAR-iCLIP sequencing libraries for Imp. A representative autoradiograph of both types of RNA–Imp cross-linking complexes used for library generation is depicted in Figure S2a in Additional file 1, and the mapping statistics of the sequenced reads are summarized in Figure S2b in Additional file 1. We confidently mapped 37–50 % of the reads (2.1–4.7 × 10^6^) to the *Drosophila* genome, demonstrating the high quality of the produced libraries.

**Fig. 2:**
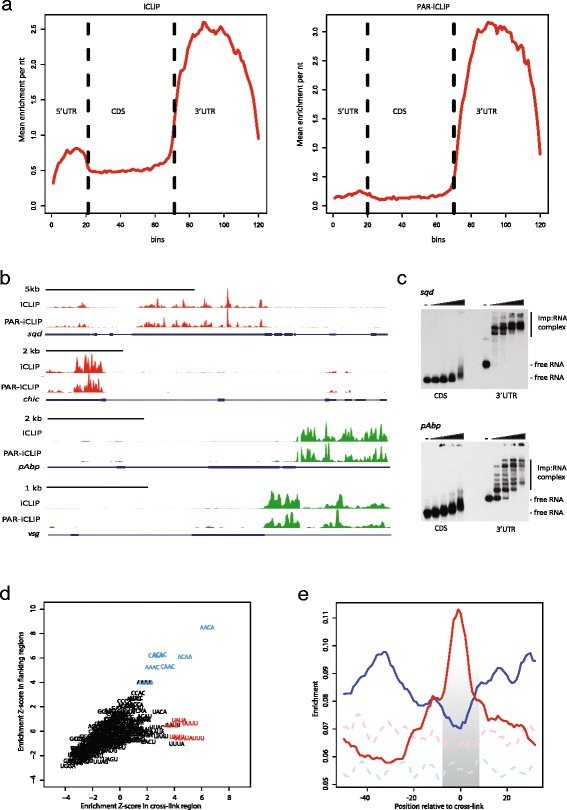
Imp CLIP identifies CA-rich binding motifs in 3′ UTRs. **a** Standardized transcript profile showing the CLIP enrichment from short-wave (*left panel*) and long-wave (*right panel*) cross-linking approaches. The y-axis displays the mean enrichment per nucleotide in bins across the 5′ UTRs (20), coding regions (*CDS*; 50) and 3′ UTRs (50) on the x-axis. **b** UCSC genome browser tracks of normalized CLIP-tags on *squid* (*sqd*), *chickadee* (*chic*), *pAbp*, and *visgun* (*vsg*) primary transcripts (*red* depicts tags derived from minus-strand transcripts and *green* those from plus-strand transcripts). The *blue boxes* correspond to the coding region, whereas UTRs and introns are depicted with *thick blue lines* and *thin blue lines*, respectively. **c** Electrophoretic mobility-shift assay of RNA segments from the coding region and 3′ UTR of the *sqd* transcript (*upper panel*) and the *pAbp* transcript (*lower panel*) with recombinant *Drosophila* Imp protein (12.5 nM, 25 nM, 50 nM, and 100 nM). **d** Scatter plot displaying the Z-score of all tetramers in the cross-link region (x-axis) and flanking regions (y-axis) in the 3000 most enriched iCLIP clusters compared with background clusters. The words with the highest enrichment either in the cross-link region or in the flanking regions are highlighted in *red* (UAUA, AUAU, AUUU, UAUU, UUAU, UUUU) and *blue* (ACAC, CACA, AACA, ACAA, CAAA, AAAC, CAAC), respectively. **e** Plot of the running mean (over five nucleotides) of positional enrichment, showing the distribution of the CA-rich (*blue line*) and UA-rich (*red line*) motifs identified in panel (d) in highly enriched iCLIP clusters. The shaded area highlights the cross-link region. Enrichment of the same motifs in randomly selected (background) clusters from 3′ UTRs is depicted by the *dashed light blue line* (CA-rich motifs) and *pink line* (UA-rich motifs)

In order to quantify the strength of Imp binding and eliminate the contribution from RNA expression, the Imp iCLIP and PAR-iCLIP tag clusters were normalized to transcript levels determined by RNA-seq. To provide a global view of Imp RNA-binding specificity we analyzed the average distribution of tag clusters across mRNA regions, including the 5′ UTR, coding sequence (CDS) and 3′ UTR (Fig. 2a). The CLIP tag clusters predominantly map to the 3′ UTR, showing a striking preference for Imp binding to this region regardless of the chosen procedure. In addition, the average distribution profile revealed that a higher 3′ UTR:CDS ratio in terms of normalized Imp CLIP tags was observed with the long wavelength PAR-iCLIP approach, reflecting the lesser background observed in the autoradiograph in Figure S2a in Additional file 1.

Imp binding frequently covered the entire 3′ UTR with peaks and valleys in the tag clusters, as shown here for *squid*, *chickadee*, *pAbp* and *visgun* transcripts (Fig. 2b). To assess the reproducibility of the experiments, the correlation of the total enrichment values, based on CLIP tags per nucleotide in 3′ UTRs normalized to the average RNA-seq expression, was evaluated between the two iCLIP libraries and between the two PAR-iCLIP libraries (Figure S2c in Additional file 1). There was a high correlation (R = 0.74 and R = 0.74 among iCLIP and PAR-iCLIP libraries, respectively) and, more importantly, a similar high correlation (R = 0.76) between the enrichment values obtained using iCLIP and PAR-iCLIP procedures (Figure S2c in Additional file 1).

Electrophoretic mobility-shift assays of *squid* and *pAbp* 3′ UTR segments, which exhibited efficient UV cross-linking in vivo, confirmed the direct binding of recombinant Imp protein to target RNAs in vitro (Fig. 2c; Figure S2i in Additional file 1). Moreover, the absence of Imp interaction with coding regions in vivo was confirmed by electrophoretic mobility-shift assay in vitro. At increasing concentrations of Imp we observed an oligomerization pattern of recombinant Imp on individual 3′ UTR segments, suggesting formation of higher-order RNP complexes. In contrast to what was observed for the predominantly 3′ UTR-binding human antigen R (HuR) RBP, the number of Imp iCLIP tags per nucleotide correlated with 3′ UTR length (Figure S2d in Additional file 1), thus corroborating the oligomerization behavior of Imp on its RNA targets on a global scale. Taken together, the in vivo cross-linking and in vitro binding experiments imply that 3′ UTRs provide efficient Imp binding platforms, whereas this is not the case for coding regions.

In order to obtain insight into the motif recognized by Imp, we merged the two replicates for each iCLIP protocol and carried out motif analyses using cWords [24] on 25,000 clusters, ranked by the maximal CLIP enrichment of each cluster. The top 10 tetramers obtained from these analyses are depicted in Figure S2e in Additional file 1. We found a significant enrichment of CA-rich motifs, mainly as CA and CAA repeats, and UA-rich motifs in both datasets. The co-occurrence analysis of MACA motifs (M designates C or A) in highly enriched CLIP clusters showed fluctuations following a periodic pattern, confirming the repetitive structure of these motifs in the CLIP clusters (Figure S2f and “Supplementary Materials and methods” in Additional file 1).

Crystallographic studies of single KH domain–nucleic acid interactions such as Nova-2 KH3 [25] and hnRNP K KH3 [26] suggest that KH domains recognize tetranucleotides situated in a molecular vise between the GxxG signature loop and the variable loop. Therefore, we analyzed the positional enrichment of all tetramers in the cross-link region defined by a high occurrence of iCLIP read starts [18]. Figure 2d shows the Z-score of tetramers in the 3000 most enriched iCLIP clusters in 3′ UTRs compared with randomly selected clusters from 3′ UTRs (background) in the cross-link region (x-axis) and the flanking regions (y-axis). The scatter plot identifies a group of UA-rich words that are highly enriched in the cross-link region whereas the CA-rich words are mainly enriched in the flanking regions. The positional enrichment of these motifs in the vicinity of iCLIP clusters is depicted in Fig. 2e. We observe a strong enrichment of the UA-rich words around the beginning of clusters (grey shaded area) whereas CA-rich motifs appear mainly in the flanking regions. Similar results were also found for PAR-iCLIP (Figure S2g, h in Additional file 1), although a 4-thiouridine-mediated bias towards U-rich motifs was observed (Figure S2e in Additional file 1). We conclude that *Drosophila* Imp has a specific positional sequence preference that extends over distances longer than a single tetramer motif.

### Imp binds transcripts important for correct neural development and reproduction

**Fig. 3:**
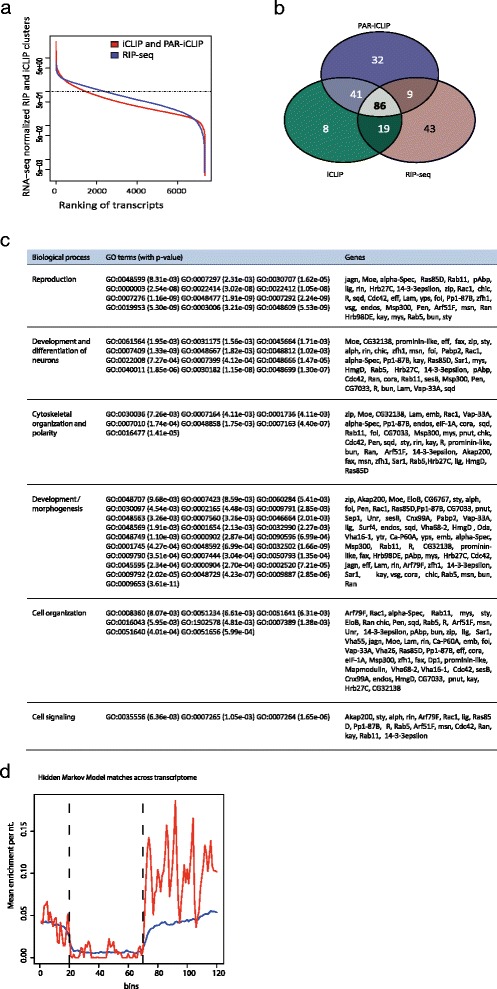
Imp RNPs contain transcripts associated with neural development and reproduction. **a** Enrichment of combined iCLIP and PAR-iCLIP clusters (*red curve*) and RIP-seq data (*blue curve*) across the Imp targetome. On the x-axis, the transcripts are ranked according to the enrichment of the individual transcript shown on the y-axis. The horizontal dotted line at y-value 1 indicates equal relative amounts of CLIP clusters or RIP-seq reads and RNA-seq levels. **b** Venn diagram of highly ranked transcripts derived from six individual experiments utilizing three distinct experimental approaches. In each experiment the 200 most highly ranked transcripts are identified, and the common pool for each experimental approach is represented by the three ellipses (157 for RIP-seq, 154 for iCLIP, and 168 for PAR-iCLIP) **c** GO term analyses of the 86 transcripts present among the 200 most highly ranked transcripts in each of six individual experiments utilizing three distinct experimental approaches and a *P* value cutoff of 0.01. Figure S3b in Additional file 1 lists the protein family or conserved domains encoded by each of the 86 *Drosophila* transcripts. **d** Standardized transcript profile showing the average number of motifs per nucleotide predicted with the hidden Markov model. The y-axis represents the average motif counts per nucleotide and the x-axis shows the position of the motif along the standardized transcript. The *red line* indicates the number of matches in the top 86 transcripts identified by iCLIP, PAR-iCLIP and RIP-seq datasets. The *blue line* displays the average motifs in all transcripts with a least 30 % RNA-seq coverage. Vertical dashed lines indicate borders between UTRs and CDS

In order to assess the transcriptome-wide Imp association, we examined the CLIP and RIP-seq enrichment of all expressed transcripts in the S2 cell transcriptome (Fig. 3a; “Supplementary Materials and methods” in Additional file 1). In both cases, the profiles showed that many transcripts exhibited some degree of association with Imp. However, a subpopulation of about 200 transcripts displayed a more than three-fold enrichment compared with RNA-seq.

To define enriched Imp targets in a conservative manner, the intersection between the top 200 targets identified by iCLIP, PAR-iCLIP and RIP-seq was selected, resulting in 86 transcripts common to all six experiments (Fig. 3b). Enrichment of both iCLIP and PAR-iCLIP clusters among the subpopulation of highly ranked transcripts (red curves in Figure S3a in Additional file 1) was above that exhibited by the whole transcriptome (blue curves in Figure S3a in Additional file 1).

The 86 shared transcripts, as well as their protein family association, are ranked in Figure S3b in Additional file 1 according to their average enrichment, and GO term enrichment analysis was performed on the 86 top targets found in all datasets. Significant enrichment for GO categories representing biological processes such as reproduction, neural development and cell polarity was observed (Fig. 3c).

To examine the enrichment of the previously identified Imp binding motif among the 86 top ranked transcripts, we designed a hidden Markov model (HMM) that models the CA-rich repeat regions and the UA-rich cross-link motif to identify Imp binding sites (see “Materials and methods”; Figure S3c in Additional file 1). As expected, we observe a two- to threefold higher enrichment of Imp binding motifs in 76 top targets (Fig. 3d, *red line*) compared with the S2 cell line transcriptome (Fig. 3d, *blue line*). An example of these binding motifs can be found in the highly enriched clusters present in *Pendulin* and *pAbp* 3′ UTRs (Figure S3d in Additional file 1).

We conclude that Imp binds to a UA-rich motif flanked by CA-rich motifs in 3′ UTRs of transcripts important for neural development and reproduction. By examining the list of the shared 86 transcripts as well as their GO term categories, we can also conclude that Imp has a strong preference for 3′ UTRs in transcripts encoding crucial components of cellular protrusion dynamics, such as Moesin, Chickadee (profilin homolog), Rho GTPases (Rac1 and Cdc42), the exchange inhibitor GDI, the 14-3-3epsilon adaptor, and Arf79f (Arf1 homolog), to name a few.

### Knockdown of Imp affects the F-actin level

The RNA interaction network described in Fig. 3c suggests that Imp may play a role in remodeling the actin cytoskeleton. To test this hypothesis we examined the effect of Imp depletion on F-actin levels and organization using phalloidin, which binds to the interface of actin subunits in F-actin. Double-stranded RNA interference (dsRNAi)-mediated knockdown of Imp was carried out in parallel with dsRNAi-mediated knockdown of firefly luciferase, the latter acting as a negative control. Since inhibition of the Rho-associated protein kinase (ROCK) with Y-27632 has been shown to result in the suppression of cofilin phosphorylation and increased severing of F-actin [27], we also examined the effect of Imp knockdown in the absence and presence of the ROCK inhibitor in S2 cells seeded on concanavalin A [28].

**Fig. 4:**
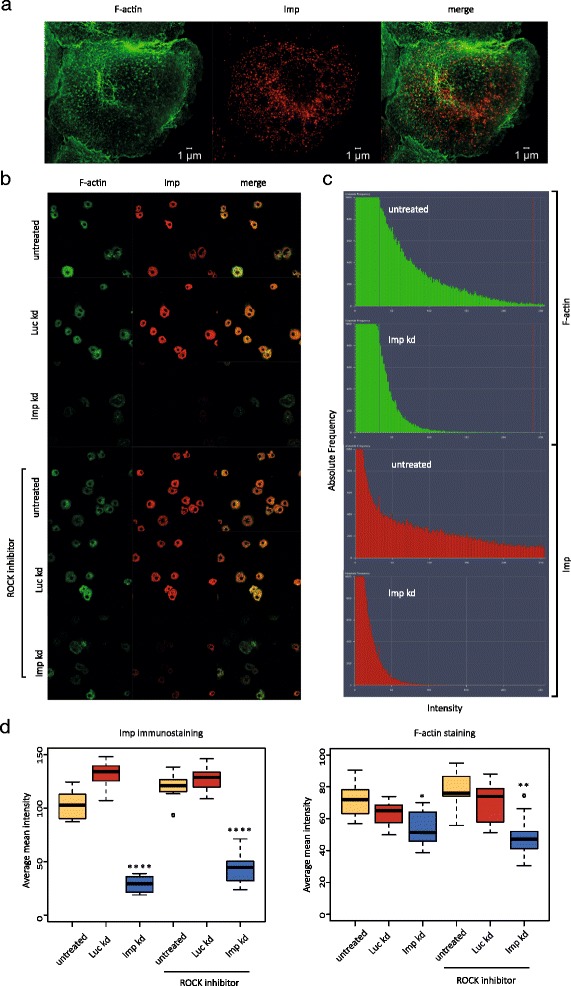
Knockdown of Imp leads to diminished F-actin levels. **a** Structured illumination of Alexa 488-phalloidin (*green*) and Alexa 568 (*red*) Imp in *Drosophila* S2 cells. **b** Selected visual fields of untreated, *luciferase* dsRNA-treated, or *imp* dsRNA-treated S2 cells, in the presence or absence of the ROCK inhibitor Y-27632. **c** The absolute frequencies of Alexa 488 and Alexa 568 pixels in a visual field of untreated S2 cells and cells with Imp knock-down, which were used to derive the average mean intensities shown in panel (d). **d** The pixel intensities of ten frames from each cell dish were measured, and the boxplots display the average mean intensity of Imp immunoreactivity (*left panel*) and phalloidin staining (*right panel*). *P* values were calculated using Student’s *t*-test. *P ≤ 0.05, **P ≤ 0.01 and ****P ≤ 0.0001

Imp immunoreactivity and phalloidin staining in an S2 cell are depicted in Fig. 4a, whereas visual fields from cell dishes subjected to various treatments are shown in Fig. 4b. Knockdown of Imp resulted in an expected decrease in Imp immunoreactivity, and the phalloidin staining was apparently also decreased in the Imp-deficient cells. In order to quantify the effect on F-actin levels, ten fields of each cell dish were analyzed and the red Alexa 568 (anti-Imp) and green Alexa 488 (phalloidin) pixels were measured using the HISTO functionality of the ZEN 2011 software package. The mean intensities in each field, after thresholding (Fig. 4c), are listed in Figure S4a in Additional file 1. When counting the phalloidin and anti-Imp stained pixels it emerged that a reduction of endogenous Imp levels resulted in significantly less F-actin staining compared with S2 cells treated with control *luciferase* dsRNA (21 % reduction, *P* value 0.04; Fig. 4d). The reduction was more pronounced in a ROCK inhibitor-stressed background (36 % reduction, *P* value 0.002), in spite of a lack of any major changes in F-actin organization upon treatment with the ROCK inhibitor in wild-type S2 cells.

Diminished F-actin staining results from an inability to assemble filamentous actin from either a reduced level of actin monomers or a lack of other factors involved in the process. In order to determine if the reduced level of Imp caused down-regulation of mRNAs participating in F-actin formation, RNA-seq from *imp* dsRNA- and *luciferase* dsRNA-treated cells were carried out. Imp knockdown resulted in a reduction of the Imp protein level to 11–21 % of the endogenous level in S2 cells (Figure S4b in Additional file 1). The biological triplicate analyses of the steady-state mRNA levels were unable to identify statistically significant changes when correcting for multiple hypotheses testing (for output of differential expression sequencing (DESeq) analysis see Gene Expression Omnibus accession number GSE62997). Since knock-down of Imp did not result in any changes at the transcript level, we investigated whether reduction of Imp caused diminished levels of actin monomers. A western blot analysis of *imp* dsRNA- and *luciferase* dsRNA-treated cell lysates was unable to detect a change of actin monomer concentration in an Imp-deficient setting (Figure S4c in Additional file 1). Therefore, we infer that the level of Imp affects the formation of F-actin rather than individual transcripts or actin monomer concentration.

### Knockdown of Imp results in abnormal neuronal patterning in embryos

Since reduction of Imp levels resulted in compromised F-actin formation in S2 cells, we hypothesized that an Imp-deficient embryo would exhibit developmental defects. Therefore, we examined the *imp*^*G0072*^ mutant strain, which exhibits decreased *imp* transcript levels and is semi-lethal due to a P-element insertion in the *imp* locus [29]. Quantitative RT-PCR analysis of a collection of pupae showed that the *imp* transcript level was reduced to 8 % of the wild-type level (data not shown). To corroborate that the semi-lethal phenotype of *imp*^*G0072*^ flies was due to a disturbance at the *imp* locus, excision of the P-element was induced and both wild-type sequence and phenotype were restored, as previously described by Geng and Macdonald [13] (Figure S5 and “Supplementary Materials and methods” in Additional file 1). Increased mortality of the *imp*^*G0072*^ progeny compared with both wild-type and an FM7c balanced/wt was observed during all stages of life. Closer examination of the eclosing flies revealed that only 12 % (n = 383) were hemizygous males compared with the expected 25 %. Moreover, we found 30 hemizygous males struggling to emerge from their pupal cases, and among the 45 hemizygous males that did manage to eclose, all drowned shortly after in the medium.

**Fig. 5:**
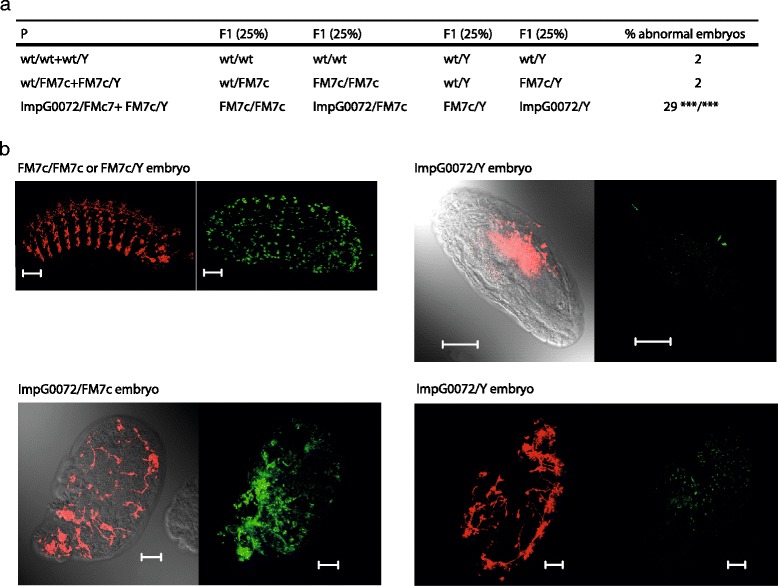
Reduced Imp levels result in aberrant neuronal patterning. **a** Theoretical genotypes and observed abnormal embryos in the F1 generation. FM7c designates an X-chromosome balancer containing a wild-type (wt) *imp* gene and *β*-*gal* gene, whereas *imp* ^*G0072*^ contains a P-element in the *imp* locus on the X chromosome. Wild type and FM7c balanced wild type exhibited equal rates of abnormal embryos. A binominal *P* value of <0.001 was calculated for the *imp* ^*G0072*^ mutant compared with either wild type or FM7c balanced wild type. Number of embryos counted: wt/wt + wt/Y cross 575 embryos, wt/FM7c + FM7c/Y cross 712 embryos, *imp* ^*G0072*^/ FM7c + FM7c/Y cross 558 embryos. **b** Whole-mount immunohistochemistry with the monoclonal antibody 22C10 staining neurofilamental Futsch (*red*), and β*-*gal (*green*) marking the X-chromosome balancer. *Top left*: an embryo with multiple wild-type Imp alleles and a nervous system that appears normal (scale bar 50 μm). *Bottom left*: an embryo with one Imp allele which is morphologically abnormal and an aberrant nervous system (scale bar 50 μm). *Top right*: an *imp* ^*G0072*^ hemizygous male embryo with a relatively normal morphology and a distinguishable central nervous system exhibiting pathfinding defects (scale bar 100 μm). *Bottom right*: an *imp* ^*G0072*^ hemizygous abnormal male embryo revealing extensive patterning defects (scale bar 50 μm)

The observation that the *imp*^*G0072*^ mutants were either incapable of emerging from their pupal cases or drowned in the medium suggests locomotion problems that could be ascribed to defects in the nervous system. This led us to investigate the neuronal patterning in *imp*^*G0072*^ mutant embryos. To identify the 25 % of the *imp*^*G0072*^ progeny that exhibited no *imp* wild-type allele, a new line from the *imp*^*G0072*^ strain was created, in which a β-galactosidase (β-gal) marker was present on the X-chromosome balancer FM7c, so that expression of β-gal would be linked to the presence of an FM7c balancer with a wild-type *imp* allele. In order to record the fraction of embryos with an abnormal neuronal pattern, the nervous system of wild-type embryos, and embryos of crosses between either wt/FM7c or *imp*^*G0072*^/FM7c females and FM7c/Y males, was visualized by enzyme-linked immunohistochemistry. Twenty-nine percent of the embryos showed an abnormal phenotype, which suggested that aside from the hemizygous males (*imp*^*G0072*^/Y), some heterozygotes (*imp*^*G0072*^/FM7c) were also affected by the P-element insertion. The binominal *P* values of *imp*^*G0072*^ embryos compared with either wild-type or wt/FM7c embryos were calculated, showing significantly increased rates of embryos with abnormal morphology and nervous systems (Fig. 5a).

To examine the neuronal patterning of mutant embryos, whole-mount immunostaining of the *imp*^*G0072*^/FM7c + FM7c/Y resulting embryos, using antibodies against β-gal and the MAP1B-like protein Futsch (mAb 22C10), was carried out. Staining with 22C10 showed a large variation among the mutants, ranging from a morphologically normal looking embryo with a nervous system that at least superficially appears normal (Fig. 5b, genotype FM7c/FM7c or FM7c/Y), to a morphologically normal embryo with an underdeveloped and abnormal nervous system (Fig. 5b, genotypes *imp*^*G0072*^/FM7c and *imp*^*G0072*^/Y). We conclude that embryos with reduced Imp levels exhibit abnormal neuronal patterning in the developing nervous system, and that even survivors at the pharate adult stage have profound locomotion problems.

## Discussion

In this study, we have identified the RNA targets of the endogenous *Drosophila* Imp protein on a transcriptome-wide scale, utilizing a highly specific polyclonal antibody in six biologically independent experiments with three different methods. In general, both iCLIP and PAR-iCLIP data revealed a striking preference for direct Imp attachment to 3′ UTRs at the expense of especially the coding region. The distinct binding profile in Fig. 2a provides comprehensive evidence that the CLIP tags are not the result of random unspecific cross-linking of an abundant RBP and is also in sharp contrast to the only other published CLIP study in the S2 cell line, namely an iCLIP analysis of Ago2 in nuclear extracts that exhibited a narrower peak profile [30]. An analysis of motifs associated with Imp iCLIP clusters showed that repeated CA-rich segments provide the most likely RNA–protein interface of a composite binding site consisting of a UA-rich cross-link motif, similar to the UUUAY motif previously identified for *Drosophila* Imp using SELEX [11], flanked by CA-rich regions in the 3′ UTR, which are enriched considerably among the top 86 targets. The CA-rich motif is reminiscent of the CAUH motif reported to be involved in binding of *Drosophila* Imp to the *unpaired* 3′ UTR [22]; the CAUH motif is also recognized by human IGF2BP1–3 in HEK293 cells, and in 30 % of cases is repeated within 3–5 nucleotides [17]. Moreover, a bipartite recognition motif consisting of CGGAC and MCAY within 10–25 nucleotides of each other has been suggested for the human KH34 di-domain [31], although the RNA-protein interface of the full-length protein appears to be considerably more complex spatially [9].

Although both *Drosophila* Imp and its vertebrate homologs contain the characteristic four KH domains, the former oligomerizes on targets of merely 200 nucleotides (Fig. 2c) whereas the human homologs only dimerize on 100–250 nucleotide target RNAs [32]. The multimerization of the *Drosophila* isoform, presumably mediated by the C-terminal low-complexity Gln-rich domain, may have a bearing on the extensive 3′ UTR cross-linking pattern reported here. In fact, there is a positive correlation between the average CLIP enrichment per nucleotide and the length of the 3′ UTR, which was not observed in CLIP experiments with other RBPs that also show a strong 3′ UTR enrichment, such as HuR [33] (Figure S2f in Additional file 1), implying that *Drosophila* Imp preferentially binds transcripts with long 3′ UTRs. This is a hallmark of cooperative multimerization but additional explanations are definitely feasible in a competitive in vivo scenario. Interestingly, hydrogels derived from low-complexity RBPs exhibit a preponderance to incorporate mRNAs with long 3′ UTRs [34], and since Imp encompasses a low-complexity Gln-rich C-terminus, the biochemically identified RNA assemblage that appears “granular” in Fig. 1b may actually be hydrogels, since a biotinylated isoxazole derivative is able to precipitate Imp from S2 cells [35].

**Fig. 6:**
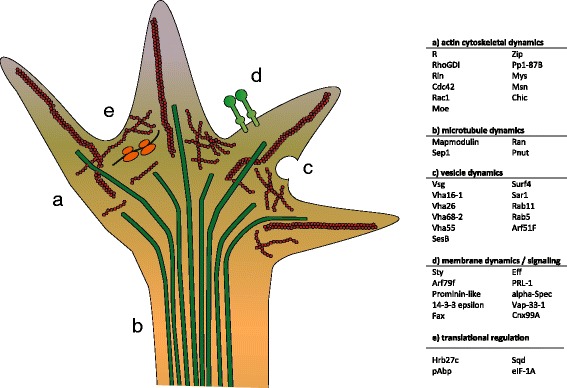
A model of the Imp post-transcriptional RNA assemblage in growth cone dynamics. Forty out of the 86 transcripts identified by the three different high-throughput analyses as being associated with Imp (Fig. 3; Figure S3b in Additional file 1) have been divided into five categories (a–e) participating in growth cone dynamics. References for each of the categorized transcripts and their involvement in neuronal development and growth cone biology are in Figure S6 in Additional file 1

The most striking result of our study is the identification of transcripts mediating actin cytoskeletal dynamics. An analysis of the RIP-seq, iCLIP and PAR-iCLIP data revealed 86 transcripts being top Imp targets based on their enrichment in both immunoprecipitated RNA and CLIP tags over transcript levels in the S2 cell line. Forty of these transcripts can be grouped into cellular processes linked to growth cone steering and axonal branching during neuronal development as illustrated in Fig. 6. The Imp RNP contains transcripts encoding the transmembrane protein Prominin-like, which is able to respond to external signals, and Cnx99A (calnexin), which is able to alter the internal Ca^2+^ environment, vital for regulation of growth cone outgrowth [36]. The GTPases (Arf79f, Cdc42, Rac1, R (Rap1) and Ran) are needed to initiate intracellular events, resulting in a reorganization of the actin cytoskeleton, while Moesin and alpha-Spectrin provide a link between the membrane and the cytoskeleton. The main instigator of the polymerization of G-actin into F-actin (Chickadee/profilin), as well as the microtubule-associated protein Mapmodulin, are also present. Transcripts encoding proteins necessary for intracellular trafficking in the growth cone (Rab5, Rab11, Arf51F and Surf4) are also found in the Imp RNP. Additional references to Fig. 6 are listed in Figure S6 in Additional file 1. Less conservative exclusion criteria reveal *enabled*, *Myosin light chain cytoplasmic*, and *Act42a* transcripts as additional Imp targets. Interestingly, the major actin isoform in S2 cells encoded by *Act42a* mRNA was present among the top 140 transcripts in all four CLIP experiments, but exhibited a low enrichment factor in the RIP experiments. This indicates that Imp binds with modest affinity to *Act42a* mRNA and, therefore, is washed away, at least to some extent, during the immunoprecipitation.

The concept of Imp homologs being involved in growth cone turning has been shown previously, but focus has been on the β-actin/ZBP1 paradigm and not on the growth cone transcriptome [37, 38]. However, a recent study of γ-neurons in mushroom bodies of *Drosophila* Imp knockout flies has shown that Chickadee/profilin overexpression is able to partially rescue a neurite branching defect [15], providing a glimpse of a more composite Imp-regulated growth cone targetome. An intriguing result of the Imp top target analysis was the absence of transcripts encoding Twinstar (the cofilin homolog responsible for severing F-actin), Lim kinase 1 (which inactivates Twinstar), and the serine-3 phosphatase Slingshot (which activates Twinstar) [39]. In an Imp-deficient background, there was a down-regulation of F-actin levels, and this phenotype was more pronounced in the presence of a ROCK inhibitor, lending support to a scenario where the Imp RNA assemblage may be coordinating F-actin polymerization. Inhibiting the function of ROCK causes increased severing of F-actin filaments, but F-actin staining was similar to that seen in untreated cells. This is most likely due to the increased presence of barbed ends and an increased potential for addition of actin monomers to the filaments. Knockdown of Imp diminished the level of F-actin, and this phenotype was more pronounced in the presence of ROCK inhibitor, suggesting that Imp is involved in the addition of actin monomers to the filaments. Since knockdown of Imp did not affect the level of actin monomers but caused a decrease in F-actin staining, we infer that the presence of the Imp assemblage facilitates F-actin polymerization.

One could speculate that the Imp RNP encapsulates transcripts needed for coordinated on-site (de novo) protein synthesis of filopodia components in response to directional guidance cues. This interpretation is supported by experiments performed in mouse primary cortical neurons [40], *Drosophila* mushroom body γ-neurons [15], and *Xenopus* retinal ganglion cells [41] that all find Imp responsible for correct branching events rather than being essential for default neurite extension.

The embryo phenotype of Imp knockdown we report in this study exhibited a broad range of penetrance — almost to the point of being stochastic — and the few survivors reaching the pharate adult stage showed severe locomotion defects. In a more general fashion, the broad range of Imp knockout phenotypes suggests that Imp provides robustness to cytoplasmic post-transcriptional regulation by supplying RNA assemblages coordinating spatiotemporal protein production essential for cellular extensions. In the same vein, during spermatogenesis Imp exhibits abundant expression in the tail cyst cell encasing the extraordinarily long spermatids [12], and its elongation is dependent on F-actin formation, so the top-ranked transcripts identified in the present study may therefore also participate in cyst cell elongation via an Imp-facilitated mechanism. Moreover, the clustering of transcripts encoding RNA-binding proteins such as Hrb27c, Squid, Ypsilon schachtel and Pabp2, combined with a critical role of the cortical actin-binding protein Moesin in *oskar* mRNA anchoring during oogenesis [42], suggests a coordinating role of Imp in the interplay between the localization apparatus and F-actin at the posterior oocyte cortex.

## Conclusions

Neurogenesis and gametogenesis are characterized by a requirement for plasticity in terms of local cellular elongation, yet preserving a differentiated or totipotent state, respectively. Therefore, coordination of gene expression has to some extent become an RNA-centric task, and the physical manifestation of the post-transcriptional RNA regulon is provided by cytoplasmic RNP granules/hydrogels encompassing functionally related mRNAs associated with similar RBPs, as shown for Imp in the present study. These RNA assemblages facilitate fast sub-cytoplasmic recruitment of genetic information to the translational apparatus, thereby ensuring synchronous on-site translation in response to extracellular cues.

## Materials and methods

### *Drosophila* cell culture

S2 cells (Invitrogen) were cultured at 26 °C in Schneider’s *Drosophila* medium (Biowest) containing 10 % heat-inactivated fetal bovine serum, 100 U/ml penicillin and 100 μg/ml streptomycin.

### Immunofluoresence of S2 cells

S2 cells were fixed on concanavalin A (Sigma-Aldrich) coated glass bottom dishes with 10 % formaldehyde for 10 min and permeabilized with 0.1 % Triton X-100 in phosphate-buffered saline (PBS) for 3 min [43]. After blocking with 1 % normal goat serum in PBS for 1 h at room temperature, cells were incubated overnight at 4 °C with polyclonal antibody raised against Imp [10]. Following washes in PBS, cells were stained with Alexa Fluor® 568 anti-rabbit antibodies (Life Technologies) and Alexa Fluor® 488 phalloidin (Life Technologies) in 1 % normal goat serum in PBS for 1.5 h at room temperature.

### Immunofluoresence of Imp dsRNAi-mediated knockdown in S2 cells

In a 6-well plate, 1 × 10^6^ cells/ml were soaked with 40 μg/ml dsRNA corresponding to the *imp* or *luciferase* genes. Each day, cells received a boost of 40 μg/ml dsRNA. After the boost on the third day, cells were treated with 10 μM ROCK inhibitor (Y-27632, Sigma Aldrich) for 20 h. The medium was removed and cells were fixed and immunostained as previously described. Cells were examined by structured illumination (SIM) and by confocal microscopy (Zeiss LSM 780) to obtain high resolution pictures and quantification of Imp and F-actin, respectively. Employing a 40× objective, Alexa 568 and Alexa 488 pixels were quantified in ten visual fields randomly selected using the automatic controller of the microscope. Pixels were quantified employing the HISTO functionality of the ZEN 2011 software package (Carl Zeiss). Channels were evaluated separately, and the mean intensity in each field was determined after thresholding. Significant differences were analyzed by a Student’s *t*-test. For more information see “Supplementary Materials and methods” in Additional file 1.

### RIP-seq

S2 cells were lysed in 50 mM Tris–HCl (pH 8.0), 100 mM NaCl, 1 % NP-40, 1.5 mM EDTA, complete EDTA-free protease inhibitor cocktail (Roche), 1 U/μl RiboLock (Thermo Scientific) and immunoprecipitated using polyclonal anti-Imp antibody coated Protein A Dynabeads (Life Technologies). Following repeated washes in lysis buffer, TRI Reagent (Sigma-Aldrich) was added to the beads and the RNA was isolated according to the manufacturer’s specifications. RNA from two biologically independent RIP-seq experiments as well as poly(A)-enriched RNA from cells isolated on the same day as cells used for RIP-seq, were fractionated, and library preparation was performed as described by [44], with the final library being amplified with 28 cycles of PCR.

### iCLIP and PAR-iCLIP sequencing

Imp iCLIP and Imp PAR-iCLIP were performed in biological replicates essentially as described in [17, 23]. Briefly, 5 × 10^7^*Drosophila* S2 cells were used for each iCLIP and PAR-iCLIP experiment. Cells for iCLIP were irradiated with 250 mJ at 254 nm UV light, whereas cells were treated with 100 μM 4-thiouridine for 16 h prior to UV cross-linking with 250 mJ at 365 nm in the PAR-iCLIP procedure. Cells were lysed with lysis buffer (50 mM Tris–HCl, pH 8.0, 100 mM NaCl, 1 % NP-40, complete EDTA-free protease inhibitor cocktail (Roche)), followed by ribonuclease T1 digestion (Thermo Scientific; 0.005 U/μl for iCLIP and 0.05 U/μl for PAR-iCLIP) in the presence of 0.0025 U/μl DNase I (Thermo Scientific) for 10 min at 37 °C. Imp–RNA complexes were immunoprecipitated using rabbit anti-Imp antibody-coated Protein A Dynabeads (Life Technologies) for 2 h at 4 °C. Beads were washed three times with high-salt buffer (50 mM Tris–HCl, pH 7.4, 500 mM NaCl, 1 % NP-40, 1 mM EDTA, 0.125 % SDS) and twice with wash buffer (20 mM Tris–HCl, pH 7.4, 10 mM MgCl_2_, 0.5 % NP-40). The RNA purification and library generation were performed as described in [23] with 21–34 cycles of PCR amplification. E-gel size selection was done in order to remove the reverse transcription and Illumina primer annealing by-product.

### RNA-seq for normalizing iCLIP data

In order to normalize iCLIP and PAR-iCLIP data, RNA-seq was performed on cells harvested on the same day as cells used in the iCLIP procedure. For each replicate, total RNA was isolated from 5 × 10^7^ cells using the standard TRI Reagent protocol (Sigma Aldrich). Poly(A) RNA was enriched from 100 μg total RNA using Poly(A) Purist MAG kit (Ambion) according to the manufacturer’s specifications. The poly(A) RNA was fragmented into approximately 280 nucleotides by heating at 95 °C for 3.5 min in the presence of 50 mM Tris–HCl (pH 8.0) and 5 mM MgCl_2_. Treatment of the RNA with T4 polynucleotide kinase (Thermo Scientific), according to the manufacturer’s specifications, resulted in dephosphorylation of the 3′ ends of the RNA. The remaining part of the RNA-seq library preparation was performed as described in [23] with 17 cycles of PCR amplification.

### Gene ontology terms

The transcripts present in a top 200 ranking of normalized RIP-seq datasets were used as input for the term enrichment tool AmiGO [45] using a *P* value cutoff of 0.0001. As background sample, a list was made of all poly(A) RNAs present in the S2 cell line according to the RNA-seq.

### Whole mount immunostaining of embryos

*Imp*^*G0072*^ was obtained from the Bloomington *Drosophila* Stock Center (stock number 11798, w67c23 P{lacW}ImpG0072/FM7c). The strain was generated by a P-element insertion in the *imp* locus in a region corresponding to an intron upstream of the initiation codons [29]. Embryos were dechorionized for two minutes in 2 % sodium hypochlorit and fixed in 4 % paraformaldehyde. In order to record the fraction of embryos with an abnormal neuronal pattern, the nervous system of wild-type embryos, and embryos of crosses between either wt/FM7c or *imp*^*G0072*^/FM7c females and FM7c/Y males, was visualized by enzyme-linked immunohistochemistry. The monoclonal neuronal marker 22C10, which recognizes the MAP1B-like protein Futsch and labels both the central and peripheral nervous systems [46], was used as the primary antibody, followed by anti-mouse horse radish peroxidase-catalyzed diaminobenzidine oxidation (Cell Signaling). The frequency of embryos with an abnormal morphology and nervous system was recorded, and the percentage of abnormal embryos was determined. In the co-staining with anti-β-gal antibody (Rockland; 1:10,000) and monoclonal anti-Futsch 22C10 antibody (1:100; Developmental Studies Hybridoma Center, University of Iowa), the secondary antibodies were anti-rabbit Alexa Fluor® 488 and anti-mouse Alexa Fluor® 555 from Molecular Probes (1:1000), respectively. Fluorescent immunostainings were visualized with a confocal Zeiss LSM510 microscope.

### Read mapping and processing

All sequencing data were produced by The Danish National High-Throughput DNA Sequencing Centre using Illumina HiSeq and 50–100 bp single-end reads. The mapping procedure for iCLIP, PAR-iCLIP, RIP and RNA-seq controls is described in “Supplementary Materials and methods” in Additional file 1.

### CLIP and RIP normalization

The iCLIP and RIP libraries were prepared from the same biological sample as that used for the RNA-seq data sets. Therefore, we normalized each RIP and iCLIP replicate with its corresponding RNA-seq replicate. The PAR-iCLIP replicates were normalized to a pool of two iCLIP RNA-seq controls. The enrichment *e* of CLIP in a position *i* of a particular cluster *k* is calculated as:

1 $$ {e}_{i,k}=\frac{M.{L}_g.{c}_i}{N{\displaystyle {\sum}_{j=1}^{L_g}}{r}_j\ } $$

where *c*_*i*_ is the count of CLIP base calls in position *i*, and *N* the total amount of confidently mapped CLIP reads. As *r*_*j*_ is the count of RNA-seq reads, $\frac{1}{M{L}_g}\left({\displaystyle \sum_{j=1}^{L_g}}{r}_j\right)$ is the sum of RNA-seq base calls across the transcript *g*, normalized to the total amount of confidently mapped RNA-seq reads *M* and the length of the transcript *L*_*g*_.

### Standardized transcript profiles

To make standardized profiles, the longest protein-coding transcript of each gene with at least 30 % RNA-seq coverage in the S2 cell line was used. The regions (5′ UTR, CDS, 3′ UTR) of those transcripts were divided into a fixed number of bins as follows: 20 bins for 5′ UTRs, 50 bins for CDS and 50 bins for 3′ UTRs. The calculation of mean enrichment is described in “Supplementary Materials and methods” in Additional file 1.

### Transcriptome-wide Imp binding and transcript ranking

To quantify *Drosophila* Imp binding across the whole transcriptome (Fig. 3a), we pooled CLIP replicates and RIP-seq replicates separately, and the CLIP was normalized by a pool of RNA-seq replicates associated with the iCLIP while the RIP was normalized to the RNA-seq associated with the RIP-seq. Transcripts were then ordered according to decreasing enrichment. Identification of transcripts bound by Imp was accomplished by calculating a normalized enrichment per nucleotide in 3′ UTRs for the iCLIP and PAR-iCLIP data and RIP-seq data. Each replicate was normalized by a pool of RNA-seq replicates associated with the iCLIP and rankings were summarized and merged. Only the 1157 most expressed transcripts with an expression level higher than the average expression of a given dataset were considered. This was done to avoid a high degree of variation due to low gene expression and thereby inconsistent rankings across replicates. This normalized coverage was then used to rank transcripts according to enrichment (Fig. 3b).

### Word enrichment analysis

Imp CLIP-seq data form very large clusters that cover the entire 3′ UTRs in some cases. Thus, upon normalization of clusters from pooled iCLIP and pooled PAR-iCLIP replicates, they were divided into smaller regions based on the enrichment changes across the clusters. In this way, segments that were highly covered by CLIP-data, i.e., highly enriched, and segments less enriched could be separated. We extracted the sequences and ranked them by maximal normalized enrichment in the associated cluster (described in Eq. 1) after discarding small clusters (less than six reads) and genes with low expression levels (average of less than five reads per nucleotide), and selected the 25,000 most enriched clusters in 3′ UTRs. These sequences and the enrichment ranking were subjected to the motif discovery method cWords [24] in two separate analyses, one for each iCLIP protocol.

### Hidden Markov model

To assess the presence of putative *Drosophila* Imp binding sites, we specified a HMM in the anHMM framework [47]. A state diagram of the HMM is shown in Figure S3c in Additional file 1. In the state diagram, all transitions are uniformly assigned, except the transition marked by the *t1* label, which is 0.95. All states have emission probabilities of the letter shown in the diagram set to 0.9. The HMM was used to identify independent instances of the CA-rich and UA-rich motifs and post-processed such that alternating occurrences of the two motifs were merged into one composite motif if they were spaced a maximum of 50 nucleotides apart. All other single motif occurrences were filtered out. We annotated the entire genome on both strands with matches using Viterbi decoding. We summarized binding sites in the top 86 transcripts bound by Imp and in all expressed transcripts with at least 30 % RNA-seq coverage in S2 cells. We found matches in 3′ UTRs for 76 of the top 86 transcripts.

### Data access

All the data reported in this paper can be found in the Gene Expression Omnibus database with accession number GSE62997.

## Additional file

Additional file 1: Supplemental Figures S1–S6 and Supplemental Materials and methods and Supplemental References. Figure S1 is related to Fig. 1, Figure S2 is related to Fig. 2, Figure S3 is related to Fig. 3, Figure S4 is related to Fig. 4, Figure S5 is related to Fig. 5, and Figure S6 is related to Fig. 6.

## Abbreviations

β-gal: β-galactosidase
CDS: coding sequence
CLIP: cross-linking and immunoprecipitation
dsRNAi: double-stranded RNA interference
GO: gene ontology
HMM: hidden Markov model
HuR: human antigen R
iCLIP: individual-nucleotide resolution cross-linking and immunoprecipitation
KH: K homology
PAR-CLIP: photoactivatable ribonucleoside cross-linking and immunoprecipitation
PBS: phosphate-buffered saline
RBP: RNA-binding protein
RIP: RNA-binding protein immunoprecipitation
RNA-seq: high-throughput sequencing of cDNAs
RNP: ribonucleoprotein
ROCK: Rho-associated protein kinase
UTR: untranslated region

## References

[1] Gomez TM, Letourneau PC (2014). Actin dynamics in growth cone motility and navigation. J Neurochem.

[2] Jung H, Gkogkas CG, Sonenberg N, Holt CE (2014). Remote control of gene function by local translation. Cell.

[3] Keene JD (2007). RNA regulons: coordination of post-transcriptional events. Nat Rev Genet.

[4] Gerber AP, Herschlag D, Brown PO (2004). Extensive association of functionally and cytotopically related mRNAs with Puf family RNA-binding proteins in yeast. PLoS Biol.

[5] Lecuyer E, Yoshida H, Parthasarathy N, Alm C, Babak T, Cerovina T (2007). Global analysis of mRNA localization reveals a prominent role in organizing cellular architecture and function. Cell.

[6] Oleynikov Y, Singer RH (2003). Real-time visualization of ZBP1 association with beta-actin mRNA during transcription and localization. Curr Biol.

[7] Zhang HL, Eom T, Oleynikov Y, Shenoy SM, Liebelt DA, Dictenberg JB (2001). Neurotrophin-induced transport of a beta-actin mRNP complex increases beta-actin levels and stimulates growth cone motility. Neuron.

[8] Nielsen FC, Nielsen J, Kristensen MA, Koch G, Christiansen J (2002). Cytoplasmic trafficking of IGF-II mRNA-binding protein by conserved KH domains. J Cell Sci.

[9] Wachter K, Kohn M, Stohr N, Huttelmaier S (2013). Subcellular localization and RNP formation of IGF2BPs (IGF2 mRNA-binding proteins) is modulated by distinct RNA-binding domains. Biol Chem.

[10] Adolph SK, DeLotto R, Nielsen FC, Christiansen J (2009). Embryonic expression of Drosophila IMP in the developing CNS and PNS. Gene Expr Patterns.

[11] Munro TP, Kwon S, Schnapp BJ, St Johnston D (2006). A repeated IMP-binding motif controls oskar mRNA translation and anchoring independently of Drosophila melanogaster IMP. J Cell Biol.

[12] Fabrizio JJ, Hickey CA, Stabrawa C, Meytes V, Hutter JA, Talbert C (2008). Imp (IGF-II mRNA-binding protein) is expressed during spermatogenesis in Drosophila melanogaster. Fly (Austin).

[13] Geng C, Macdonald PM (2006). Imp associates with squid and Hrp48 and contributes to localized expression of gurken in the oocyte. Mol Cell Biol.

[14] Boylan KL, Mische S, Li M, Marques G, Morin X, Chia W (2008). Motility screen identifies Drosophila IGF-II mRNA-binding protein–zipcode-binding protein acting in oogenesis and synaptogenesis. PLoS Genet.

[15] Medioni C, Ramialison M, Ephrussi A, Besse F (2014). Imp promotes axonal remodeling by regulating profilin mRNA during brain development. Curr Biol.

[16] Licatalosi DD, Mele A, Fak JJ, Ule J, Kayikci M, Chi SW (2008). HITS-CLIP yields genome-wide insights into brain alternative RNA processing. Nature.

[17] Hafner M, Landthaler M, Burger L, Khorshid M, Hausser J, Berninger P (2010). Transcriptome-wide identification of RNA-binding protein and microRNA target sites by PAR-CLIP. Cell.

[18] Konig J, Zarnack K, Rot G, Curk T, Kayikci M, Zupan B (2010). iCLIP reveals the function of hnRNP particles in splicing at individual nucleotide resolution. Nat Struct Mol Biol.

[19] Macias S, Plass M, Stajuda A, Michlewski G, Eyras E, Caceres JF (2012). DGCR8 HITS-CLIP reveals novel functions for the Microprocessor. Nat Struct Mol Biol.

[20] Sanford JR, Wang X, Mort M, Vanduyn N, Cooper DN, Mooney SD (2009). Splicing factor SFRS1 recognizes a functionally diverse landscape of RNA transcripts. Genome Res.

[21] Martin G, Gruber AR, Keller W, Zavolan M (2012). Genome-wide analysis of pre-mRNA 3′ end processing reveals a decisive role of human cleavage factor I in the regulation of 3′ UTR length. Cell Rep.

[22] Toledano H, D'Alterio C, Czech B, Levine E, Jones DL (2012). The let-7-Imp axis regulates ageing of the Drosophila testis stem-cell niche. Nature.

[23] Konig J, Zarnack K, Rot G, Curk T, Kayikci M, Zupan B (2011). iCLIP–transcriptome-wide mapping of protein-RNA interactions with individual nucleotide resolution. J Vis Exp.

[24] Rasmussen SH, Jacobsen A, Krogh A (2013). cWords - systematic microRNA regulatory motif discovery from mRNA expression data. Silence.

[25] Lewis HA, Musunuru K, Jensen KB, Edo C, Chen H, Darnell RB (2000). Sequence-specific RNA binding by a Nova KH domain: implications for paraneoplastic disease and the fragile X syndrome. Cell.

[26] Backe PH, Messias AC, Ravelli RB, Sattler M, Cusack S (2005). X-ray crystallographic and NMR studies of the third KH domain of hnRNP K in complex with single-stranded nucleic acids. Structure.

[27] Song X, Chen X, Yamaguchi H, Mouneimne G, Condeelis JS, Eddy RJ (2006). Initiation of cofilin activity in response to EGF is uncoupled from cofilin phosphorylation and dephosphorylation in carcinoma cells. J Cell Sci.

[28] Rogers SL, Wiedemann U, Stuurman N, Vale RD (2003). Molecular requirements for actin-based lamella formation in Drosophila S2 cells. J Cell Biol.

[29] Peter A, Schottler P, Werner M, Beinert N, Dowe G, Burkert P (2002). Mapping and identification of essential gene functions on the X chromosome of Drosophila. EMBO Rep.

[30] Taliaferro JM, Aspden JL, Bradley T, Marwha D, Blanchette M, Rio DC (2013). Two new and distinct roles for Drosophila Argonaute-2 in the nucleus: alternative pre-mRNA splicing and transcriptional repression. Genes Dev.

[31] Patel VL, Mitra S, Harris R, Buxbaum AR, Lionnet T, Brenowitz M (2012). Spatial arrangement of an RNA zipcode identifies mRNAs under post-transcriptional control. Genes Dev.

[32] Nielsen J, Kristensen MA, Willemoes M, Nielsen FC, Christiansen J (2004). Sequential dimerization of human zipcode-binding protein IMP1 on RNA: a cooperative mechanism providing RNP stability. Nucleic Acids Res.

[33] Mukherjee N, Corcoran DL, Nusbaum JD, Reid DW, Georgiev S, Hafner M (2011). Integrative regulatory mapping indicates that the RNA-binding protein HuR couples pre-mRNA processing and mRNA stability. Mol Cell.

[34] Han TW, Kato M, Xie S, Wu LC, Mirzaei H, Pei J (2012). Cell-free formation of RNA granules: bound RNAs identify features and components of cellular assemblies. Cell.

[35] Kato M, Han TW, Xie S, Shi K, Du X, Wu LC (2012). Cell-free formation of RNA granules: low complexity sequence domains form dynamic fibers within hydrogels. Cell.

[36] Tang F, Dent EW, Kalil K (2003). Spontaneous calcium transients in developing cortical neurons regulate axon outgrowth. J Neurosci.

[37] Leung KM, van Horck FP, Lin AC, Allison R, Standart N, Holt CE (2006). Asymmetrical beta-actin mRNA translation in growth cones mediates attractive turning to netrin-1. Nat Neurosci.

[38] Yao J, Sasaki Y, Wen Z, Bassell GJ, Zheng JQ (2006). An essential role for beta-actin mRNA localization and translation in Ca2 + −dependent growth cone guidance. Nat Neurosci.

[39] Niwa R, Nagata-Ohashi K, Takeichi M, Mizuno K, Uemura T (2002). Control of actin reorganization by Slingshot, a family of phosphatases that dephosphorylate ADF/cofilin. Cell.

[40] Welshhans K, Bassell GJ (2011). Netrin-1-induced local beta-actin synthesis and growth cone guidance requires zipcode binding protein 1. J Neurosci.

[41] Kalous A, Stake JI, Yisraeli JK, Holt CE (2014). RNA-binding protein Vg1RBP regulates terminal arbor formation but not long-range axon navigation in the developing visual system. Dev Neurobiol.

[42] Jankovics F, Sinka R, Lukacsovich T, Erdelyi M (2002). MOESIN crosslinks actin and cell membrane in Drosophila oocytes and is required for OSKAR anchoring. Curr Biol.

[43] Buster DW, Nye J, Klebba JE, Rogers GC (2010). Preparation of Drosophila S2 cells for light microscopy. J Vis Exp.

[44] Kielpinski LJ, Boyd M, Sandelin A, Vinther J (2013). Detection of reverse transcriptase termination sites using cDNA ligation and massive parallel sequencing. Methods Mol Biol.

[45] Boyle EI, Weng S, Gollub J, Jin H, Botstein D, Cherry JM (2004). GO::TermFinder–open source software for accessing Gene Ontology information and finding significantly enriched Gene Ontology terms associated with a list of genes. Bioinformatics.

[46] Hummel T, Krukkert K, Roos J, Davis G, Klambt C (2000). Drosophila Futsch/22C10 is a MAP1B-like protein required for dendritic and axonal development. Neuron.

[47] Sonnhammer EL, von Heijne G, Krogh A (1998). A hidden Markov model for predicting transmembrane helices in protein sequences. Proc Int Conf Intell Syst Mol Biol.

