# Supplementary material for: Drosophila Imp iCLIP identifies an RNA assemblage coordinating F-actin formation
Source: Genome Biol. 2015 Jun 9;16(1):123. doi: 10.1186/s13059-015-0687-0 (PMC4477473; doi:10.1186/s13059-015-0687-0)
Supplement: Additional file 1: — Supplemental Figures S1–S6 and Supplemental Materials and methods and Supplemental References. Figure S1 is related to Fig. 1, Figure S2 is related to Fig. 2, Figure S3 is related to Fig. 3, Figure S4 is related to Fig. 4, Figure S5 is related to Fig. 5, and Figure S6 is related to Fig. 6. [file 13059_2015_687_MOESM1_ESM.pdf]

SUPPLEMENTAL MATERIAL

Six supplemental figures:

Supplemental Figure S1: Related to Figure 1

Supplemental Figure S2: Related to Figure 2

Supplemental Figure S3: Related to Figure 3

Supplemental Figure S4: Related to Figure 4

Supplemental Figure S5: Related to Figure 5

Supplemental Figure S6: Related to Figure 6

Supplemental Materials and methods

Supplemental References

Supplemental Figures

Hansen_SupFig_S1

A

RIP-seq replicate 1			RIP-seq replicate 2		
**All reads**		3949056	**All reads**		2411025
**Mapped**	60.1%	2371742	**Mapped**	60.1%	1448572
**Unmapped**	39.9%	1577314	**Unmapped**	39.9%	962453
**Confidently mapped**	50.2%	1982481	**Confidently mapped**	49.3%	1188113
**Multiple mapped**	9.9%	389261	**Multiple mapped**	10.8%	260459

B

Biological process	GO terms (with P-value)	Transcripts
Reproduction	GO:0000003 (2.22e-11) GO:0048477 (1.80e-10) GO:0032504 (1.82e-10) GO:0044702 (3.15e-10) GO:0003006 (3.44e-10) GO:0022412 (6.49e-10) GO:0019953 (1.80e-09) GO:0048609 (1.90e-09) GO:0044703 (3.14e-09) GO:0022414 (4.65e-09) GO:0009790 (3.40e-08) GO:0001700 (1.01e-07) GO:0009792 (1.91e-07) GO:0030707 (1.45e-06) GO:0007297 (9.39e-06) GO:0048568 (4.25e-05)	zip, Rho1, His3.3B, Moe, eff, Lam, Cortactin, oys, yps, smt3, kek1, scyl, cpa, mts,Pvf2, Ras85D, srp, Ggamma 1 , endos, Rab10, bel, R, sqd, RasGAP1, pnt, eIF-4E, Hrb27C, CycG, 14-3-3epsilon, Ran,aop, alph, sty, jagn, Pdcd4, NUCB1, Stat92E, cpb, rin, l(2)gl, Eb1,egh, foi, R ac1, lig, Pp1-87B, vsg, zfh1, ena, Rab11, 14-3-3zeta, Sar1, mys, chic, Rab5, Arf51F, msn, Hrb98DE, pAbp, bun, Hrb87F, Cdc42
Neuron	GO:0030182 (2.32e-08) GO:0048699 (3.32e-08) GO:0040011 (1.07e-05) GO:0048666 (2.14e-05) GO:0050767 (2.16e-05) GO:0051960 (2.69e-05)	zip, Rho1, Moe, Cortactin, oys, CG32138, prominin-like, eff, Not1, smt3, mts, Rip11, Ras85D, Taf4, Ggam ma1, srp, R, RasGAP1, sqd, pnt, Hrb27C, Ran, Stat92E, 14-3-3epsilon, cpb, aop, sty, Rab1, alph, rin, l(2)gl, egh, foi, fax, Rac1, Pp1-87B, zfh1, CG9705, Sar1, ena, HmgD, Rab11, 14-3-3zeta, mys, wgn, chic, Rab5, msn, pAbp, bun, Cdc42
Development and morphogenesis	GO:0009653 (3.97e-19) GO:0048513 (1.06e-12) GO:0009887 (1.41e-11) GO:0032502 (1.66e-10) GO:0044767 (2.77e-10) GO:0048856 (3.29e-10) GO:0048468 (6.87e-10) GO:0007281 (3.12e-09) GO:0022603 (5.35e-09) GO:0048869 (8.67e-09) GO:0000902 (9.99e-09) GO:0048731 (1.58e-08) GO:0030154 (1.82e-08) GO:0032989 (2.37e-08) GO:0007275 (5.27e-08) GO:0009888 (1.70e-07) GO:0050793 (2.19e-07) GO:0016331 (3.11e-07) GO:0060429 (7.38e-07) GO:0002066 (1.45e-06) GO:0002009 (3.06e-06) GO:2000026 (3.42e-06) GO:0048729 (4.79e-06) GO:0002064 (8.93e-06) GO:0048598 (9.83e-06) GO:0002520 (1.37e-05) GO:0048534 (1.37e-05) GO:0022604 (2.16e-05) GO:0045165 (2.63e-05) GO:0000904 (5.74e-05) GO:0035295 (7.37e-05) GO:0060284 (7.77e-05)	zip, Rho1, Pvf2, l(1)G0289, tmod, Moe, CG32138, atl, prominin-like, eff, Lam, Not1, Cortactin, SmD2, slik, oys, Ca-P60A, yps, smt3, EloB, emb, Arf79F, kek1, scyl, Vap-33A, cpa, akirin, mts, Rip11, Ras85D, Taf4, srp, Surf4, endos, Ggamma1, Sep1, Rab10, bel, R,CkIalpha, sqd, RasGAP1, pnt, MAPk-Ak2, eIF-4E, Hrb27C, CycG, 14-3-3epsilon, Ran, aop, crq, sty, Pdcd4, jagn, Rab1, alph, Stat92E, crol, ytr, cpb, rin, l(2)gl, egh, fax, foi, CG6767, Eb1, Rac1, Pabp2, Mlf, lig, CtBP, lolal, Pp1-87B, vsg, zfh1, CG9705, Sar1, HmgD, ena, Rab11, 14-3-3zeta, Vha16-1, Rab7, mys, pnut, wgn, chic, Rab5, Arf51F, NAT1, msn, Hrb98DE, pAbp, bun, Capr, Hrb87 F, Unr, Cdc42, Cnx99A
Sensory organ development	GO:0048749 (1.46e-06) GO:0007423 (1.46e-06) GO:0001745 (2.95e-06) GO:0001751 (3.54e-06) GO:0001654 (4.34e-06) GO:0001754 (5.34e-06) GO:0048592 (6.32e-06) GO:0090596 (6.32e-06) GO:0046530 (9.00e-06)	Rho1, Moe, eff, Lam, Arf79F, mts, Rip11, Ras85D, R,Ra sGAP1, pnt, aop, sty, jagn, alph, Stat92E, rin, l(2)gl, Eb 1, Rac1, Mlf, CtBP, ena, Rab11, 14-3-3zeta, msn, bun, Hrb98DE, pAbp , Hrb87F
Cell signaling	GO:0007264 (1.05e-09 GO:0007265 (8.52e-07) GO:0035556 (2.25e-06)	Rho1, CG8108, smt3, Arf79F, cpa, Ras85D, Rab10, R, Lk6, RasGAP1, pnt, MAPk-Ak2, 14-3-3epsilon, Ran, sty, aop,Rab1, alph, Stat92E, cpb, rin, Rac1, lig, CtBP, Pp1-87B, Rab11, 14-3-3zeta, Rab7, Rab5, Arf51F, msn, Cdc42
Cytoskeleton, migration and cell polarity	GO:0016477 (4.35e-09) GO:0048870 (6.84e-09) GO:0007163 (9.12e-09) GO:0051674 (1.64e-08) GO:0006928 (1.82e-07) GO:0007010 (2.33e-07) GO:0030036 (5.65e-07) GO:0030029 (1.62e-06) GO:0008360 (1.80e-06) GO:0090132 (1.91e-05) GO:0010631 (1.91e-05) GO:0090130 (3.46e-05) GO:0051179 (6.97e-05)	zip, Rho1, tmod , Moe, SmD2, slik, smt3, emb, Ubc6, eIF4G, mts, endos ,Pvf2, CG32138, atl, Lam, Cortactin, oys, eIF-1A , Vap-33A, cpa, Ras85D, srp, R, Ggamma1, sqd, eIF-4E, pnt, Hrb27C, 14-3-3epsilon, Ran, sty, rin, aop, jagn , Rab1, Stat92E, l(2)gl, cpb, egh, Eb1, foi, Rac1, RhoGAP18B, lig, Pp1-87B, zfh1, ena, Rab11, 14-3-3zeta , mys, pnut , wgn, chic, Rab5, Arf51F, msn, Cdc42

S1 Figure legend

(A) The figure shows the mapping statistics of each of the biological replicates of the RIP-seq libraries. Mapping was performed as described in Supplemental Materials and methods.

(B) GO-term analysis using the term enrichment tool AmiGO, on the top 200 most highly enriched transcripts shared between RIP-seq experiments. As background sample, a list of all the transcripts present in the S2 cell line, according to the RNA-seq, was made. *P*-value was set to 0.0001.

A

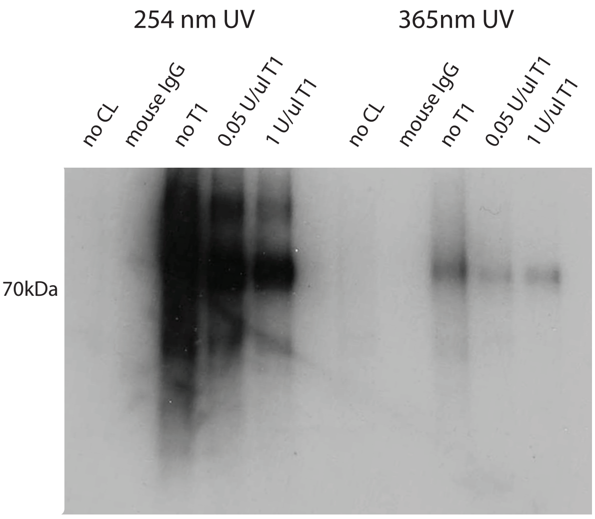

B

iCLIP 1			iCLIP 2		
All reads		2100598	All reads		4725704
Mapped	66,7%	1401138	Mapped	76,8%	3631667
Unmapped	33,3%	699460	Unmapped	23,2%	1094037
Confidently mapped	50,6%	1063906	Confidently mapped	46,3%	2188933
Multiple mapped	16,1%	337232	Multiple mapped	30,5%	1442734

PAR-iCLIP 1			PAR-iCLIP 2		
All reads		4480847	All reads		2303816
Mapped	73,3%	3282307	Mapped	67,4%	1552811
Unmapped	26,7%	1198540	Unmapped	32,6%	751005
Confidently mapped	47,6%	2132574	Confidently mapped	37,6%	866911
Multiple mapped	25,7%	1149733	Multiple mapped	29,8%	685900

C

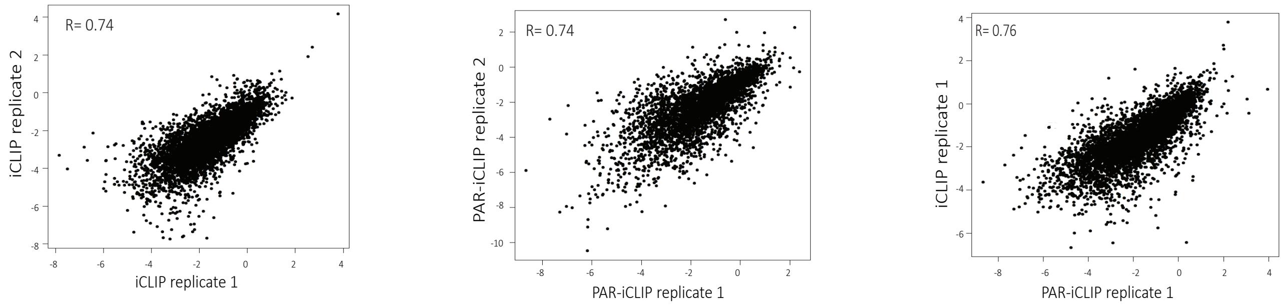

D

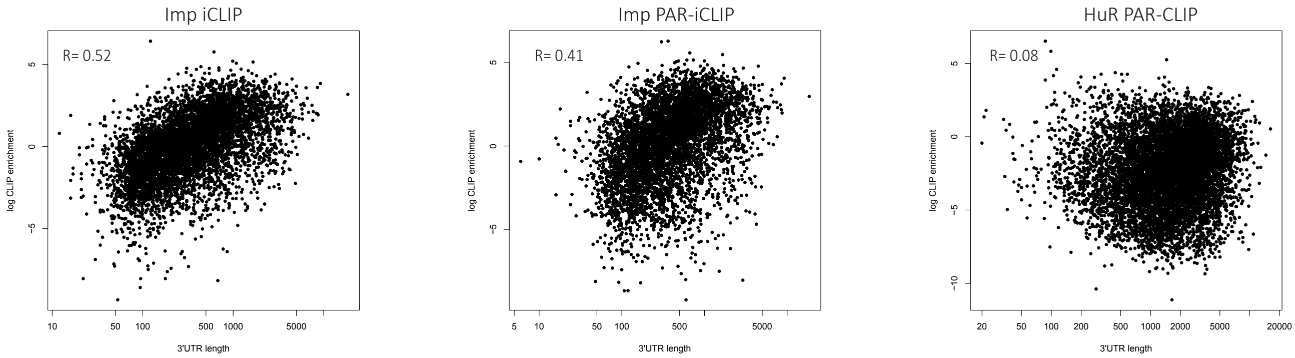

E

iCLIP		PAR-iCLIP	
CACA	43.25	UUUU	20.94
UUUU	41.33	UUUC	19.02
ACAC	40.76	UUUG	14.52
AACA	37.01	AUUU	13.73
ACAA	34.6	UUCA	13.46
CAAA	33.9	CACA	13.42
AUUU	32.55	CCAC	13.39
AAAC	29.97	GUUU	12.81
CAAC	29.75	CAAA	12.78
AAAA	29.51	UUCC	12.43

F

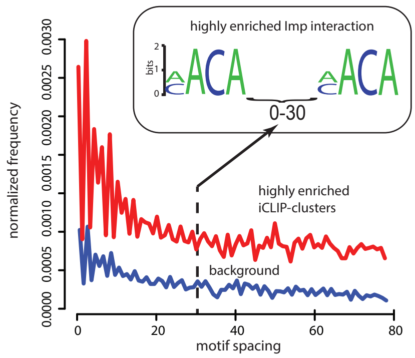

G

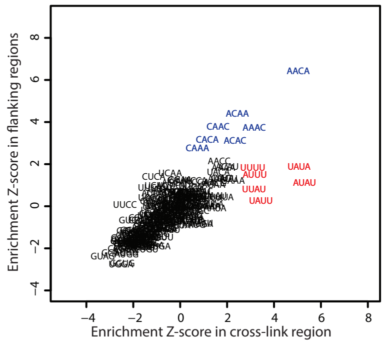

H

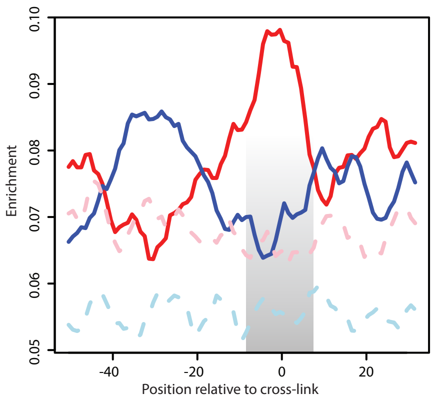

I

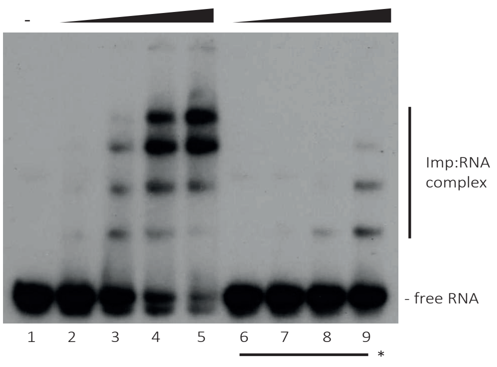

S2 Figure legend

(A) An autoradiogram of the cross-linked immunoprecipitated RNA:Imp complexes.
In both iCLIP (254nm UV cross-linking) and PAR-iCLIP (365nm UV cross-linking), the isolation of RNA:Imp complexes was dependent on cross-linking (no signal is seen in the absence of cross-linking), dependent upon Imp antibody pulldown (no signal is seen when attempting pull-down with anti-mouse IgG coated Protein A Dynabeads), and it was sensitive to RNAse T1 treatment (a higher concentration of T1 results in trimming of the RNA:Imp complexes).

(B) Mapping statistics of all four CLIP libraries

(C) Correlation analysis between the two iCLIP replicates, the two PAR-iCLIP replicates and iCLIP replicate 1 and PAR-iCLIP replicate 1. The scatter plot shows the natural logarithm of CLIP enrichment values per nucleotide in 3'UTRs for each dataset. There is a strong correlation both among the biological replicates and between protocols.

(D) Correlation analysis between CLIP tags per nucleotide and the length of the 3'UTR.
The scatterplots show a correlation between the length of the 3'UTR and the enrichment of CLIP tags per nucleotide. This phenomenon was seen for both iCLIP and PAR-iCLIP datasets. However, this correlation was not found for the RNA-binding protein HuR (HuR PAR-CLIP data obtained from [1].

(E) Top 10 most over-represented words from cWords motif analysis of top 25,000 variable sized sequences, covered by iCLIP and PAR-iCLIP clusters ranked by normalized CLIP enrichment.

(F) Relationship between co-occurrence frequency and motif spacing. The red line illustrates normalized frequency of co-occurring MACA motifs in highly enriched fixed sized sequences covered by iCLIP clusters in 3'UTRs. The blue line shows the frequency in randomly selected clusters of the same length as above from 3'UTRs. The boxed motif shows the highly enriched binding sequences and over-represented spacing distribution, with distances from 0 to 30 nt. In this plot, a horizontal line means that there is no dependence between MACA co-occurrences. We see a higher dependence when spacing between MACA occurrences is smaller than 30 nucleotides due to the presence of low-complexity regions, which is further supported by the strongly fluctuating pattern. Furthermore, we observe that in highly enriched fixed clusters co-occurrence of MACA motifs is around 4 times more frequent than in the background clusters.

(G) Scatter plot displaying the Z-score of all 4-mers in cross-link region (x-axis) and flanking regions (y-axis) in the 3000 most enriched PAR-iCLIP clusters compared to background clusters. The words with the highest Z-score either in the cross-link region or in the flanking regions are highlighted in red (UAUA, AUAU, AUUU, UAUU, UUAU, UUUU) and blue (ACAC, CACA, AACA, ACAA, CAAA, AAAC, CAAC), respectively.

(H) Plot of a running mean (over 5 nucleotides) of positional enrichment showing the distribution of the CA- (blue line) and UA-rich (red line) words identified in Supplementary Figure 2G in high-affinity PAR-iCLIP clusters. The shaded area highlights the cross-link region. Enrichment of the same motifs in randomly selected (background) clusters from 3'UTRs is depicted by dashed light blue (CA-rich motifs) and pink (UA-rich motifs).

(I) Electrophoretic mobility-shift assay of an RNA segment from the 3'UTR of the *pAbp* transcript with recombinant *Drosophila* Imp protein (2.5 nM, 7.5 nM, 22.5 nM, and 67.5 nM). The bar with the asterisk represents addition of 100 nM unlabeled target RNA.

Hansen_SupFig_S3

A

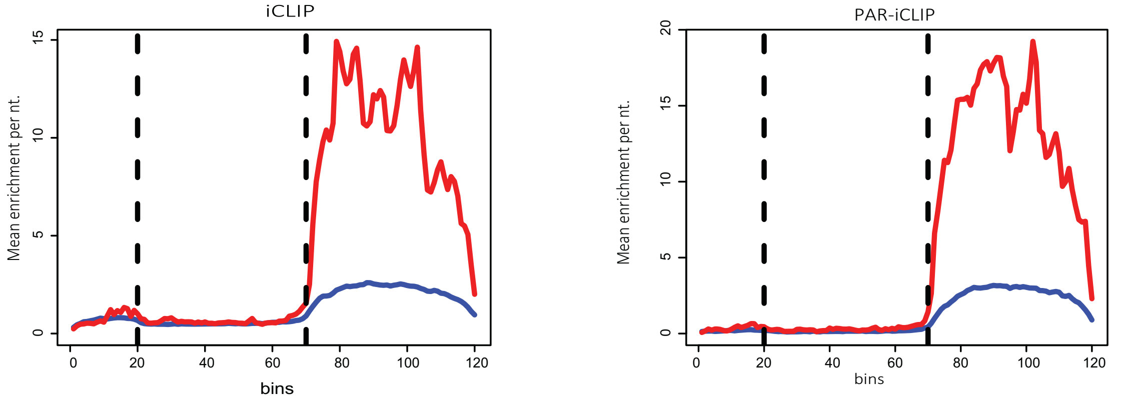

B

86 transcripts	Protein family / conserved domains	*86 transcripts*	Protein family / conserved domains	86 transcripts	Protein family / conserved domains
Msr-110		*Zip*	Myosin-II heavy chain	*alt*	aluminium tube
sqd	hnRNP	*yps*	Y-box protein	*CG32699*	LPCAT1 homolog
Mapmodulin	ANP32	*Lam*	Lamin	*Capr*	Caprin
sesB	SLC25	*Pp1-87B*	Protein phosphatase 1	*msn*	STE20 kinase
Pen	Importin-alpha 2	*CG43444*	Zinc finger/ oxygenase	*foi*	ZIP Zinc transporter domain
PRL-1	PTP family	*vsg*	Sialomucins	*HmgD*	High mobility group protein D
ytr	arginine-rich	*CG3800*	USMBP	*Unr*	S1 RNA-binding domain
Ca-P60A	Calcium-transporting ATPase	*CG9821*	PNRC domain	*sep1*	Septin
Akap200	A-kinase-anchor-protein	*CG32138*	FMNL2 domain	*Ras85D*	Ras GTPase
pAbp	Cytoplasmatic PABP	*sar1*	Small GTPase	*alph*	Protein phosphatase 2C
R	Ras family of small GTPases	*zfh1*	Zink finger homeodomain 1	*pnut*	Septin GTPase
l(1)G0320	Translocon-associated protein	*ran*	RAS oncogene family	*chic*	Profilin
CG6767	PRPS1	*Oda*	ODC antizyme	*emb*	Exportin-1
Moe	ERM family protein	*mys*	Integrin beta	*CG11357*	Glycosyl transferase
lig	UBA domain containing protein	*rin*	RasGAP SH3 binding protein	*alpha-Spec*	Spectrin
Dp1	Vigilin, HDLBP	*Cdc42*	Rho GTPase	*cora*	Protein 4.1 homolog
Vha55	Vacuolar H^+ ATPase	*D1*	AT hook-containing DNA-binding	*Vap-33-1*	VAP
Arf79F	ARF	*CG1910*		*Rab11*	Rab family GTPase
Cnx99A	Calnexin	*CG6707*	TMEM55B homolog	*Elongin-B*	Elongin BC containing E3 ubiquitin ligases
CG9281	ABCF2	*Vha68-2*	Vacuolar H^+ ATPase	*Pabp2*	Nuclear PABP
RhoGDI	Rho family GTPase	*fax*	failed axon connections	*bun*	TSC-22/Dip/BUN
eIF-1A	eIF-1A	*endos*	endosulfine	*sty*	Sprouty
Vha26	Vacuolar H^+ ATPase	*HmgZ*	High mobility group box	*kay*	Fos
Surf4	Surfeit 4	*Hrb98DE*	hnRNP	*Hsc70Cb*	Heat shock protein 70
CG7033	CCT2 homolog	*Vha16-1*	Vacuolar H^+ ATPase	*Rab5*	Rab family GTPase
eff	UBE2	*Rac1*	Rho GTPase	*CG31523*	
CG17765	PEF1 homolog	*Msp-300*	KASH	*Aps*	Nudix hydrolase
Hrb27C	hnRNP	*prominin-like*	Prominin	*Arf51F*	ADP ribosylation factor
14-3-3epsilon	14-3-3	*jagn*	ER membrane protein		

C

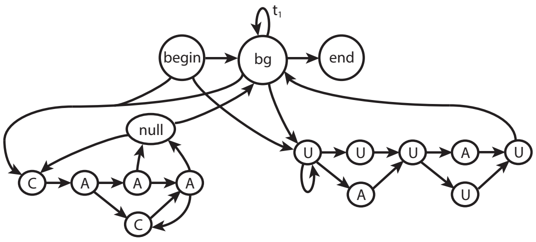

D

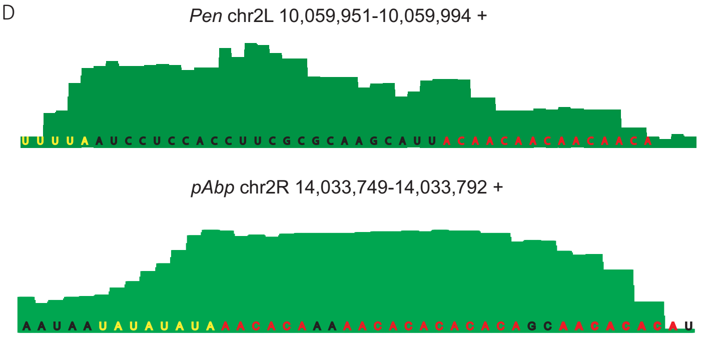

S3 Figure legend

(A) Standardized transcript profile showing the CLIP enrichment from iCLIP (left) and PAR-iCLIP (right) in the top 86 transcripts highly enriched in iCLIP, PAR-iCLIP and RIP-Seq experiments (red line) compared to the remaining transcripts expressed in S2 cells (blue line). The y-axis displays the average CLIP enrichment and the x-axis shows the position along the standardized transcript.

(B) List of the 86 shared transcripts between the RIP-seq, iCLIP and PAR-iCLIP analyses.
The NCBI Homologene database and literature search were used to identify the protein family or conserved domains of each of the 86 *Drosophila* transcripts.

(C) State diagram of the HMM designed to identify Imp binding motifs in transcripts. In the state diagram all transitions are uniformly assigned, except the transition marked by the t1 label, which is 0.95. All states have emission probabilities of the letter shown in the diagram set to 0.9.

(D) Close-up of two highly enriched iCLIP clusters along the *Pendulin (Pen)* and *pAbp* transcripts. The corresponding nucleotide sequence shows the putative Imp binding sequences highlighted. UA-rich motifs are displayed in yellow and CA-rich motifs are highlighted in red.

A

	UT Ch1	UT Ch1	UT Ch1	UT Ch1	UT Ch1	UT Ch1	UT Ch1	UT Ch1	UT Ch1	UT Ch1	LUC Ch1	LUC Ch1	LUC Ch1	LUC Ch1	LUC Ch1	LUC Ch1	LUC Ch1	LUC Ch1	LUC Ch1	LUC Ch1
Mean Intensity	84,44978	73,79027	63,25836	69,73364	58,9942	56,935	75,4561	70,25649	78,15024	90,46373	57,40855	57,57564	53,85286	63,42553	66,48961	49,93305	68,59592	68,12485	73,91709	69,47479
Standard Deviation	44,7206	40,82801	39,16477	35,60096	29,72942	33,08706	30,24877	43,01392	40,53363	44,81249	36,94278	39,08589	31,85688	37,05998	42,50451	23,31511	26,57672	43,66949	33,8971	50,31621
Pixels	48973	51275	39480	51022	40503	32292	33050	50884	51736	64212	57976	43451	41348	59455	55816	30514	33612	28074	53808	40683
Area [µm x µm]	2109,94	2209,12	1700,95	2198,22	1745,02	1391,26	1423,92	2192,28	2228,99	2766,5	2497,83	1872,04	1781,43	2561,55	2404,77	1314,66	1448,13	1209,54	2318,25	1752,78

	ROCK_UT Ch1	ROCK_UT Ch1	ROCK_UT Ch1	ROCK_UT Ch1	ROCK_UT Ch1	ROCK_UT Ch1	ROCK_UT Ch1	ROCK_UT Ch1	ROCK_UT Ch1	ROCK_UT Ch1	ROCK_LUC Ch1	ROCK_LUC Ch1	ROCK_LUC Ch1	ROCK_LUC Ch1	ROCK_LUC Ch1	ROCK_LUC Ch1	ROCK_LUC Ch1	ROCK_LUC Ch1	ROCK_LUC Ch1	ROCK_LUC Ch1
Mean Intensity	77,5549	55,81357	60,04444	74,61213	85,65893	74,13251	90,80132	94,91085	86,57809	74,51395	88,05451	64,45945	72,72229	75,54069	75,30401	51,14479	57,90086	79,00369	52,33517	80,51164
Standard Deviation	26,37495	33,06897	20,84621	32,41653	46,17716	42,84858	45,24612	46,5657	29,89838	25,55683	55,88768	30,37427	50,22105	52,37038	40,51985	28,70554	29,52379	51,14805	30,72292	46,19994
Pixels	33669	34533	26664	47944	43689	48780	62639	50927	37259	30931	46546	32850	40741	43252	46906	26895	38339	49096	31521	36913
Area [µm x µm]	1450,59	1487,81	1148,79	2065,61	1882,29	2101,63	2698,73	2194,13	1605,26	1332,63	2005,38	1415,3	1755,28	1863,46	2020,89	1158,74	1651,79	2115,24	1358,05	1590,35

	UT Ch2	UT Ch2	UT Ch2	UT Ch2	UT Ch2	UT Ch2	UT Ch2	UT Ch2	UT Ch2	UT Ch2	LUC Ch2	LUC Ch2	LUC Ch2	LUC Ch2	LUC Ch2	LUC Ch2	LUC Ch2	LUC Ch2	LUC Ch2	LUC Ch2
Mean Intensity	112,88930	108,3632	118,8245	93,61354	87,17861	90,28381	87,67987	97,19528	124,382	111,0109	107,1203	125,4367	134,7722	127,2319	133,8494	114,1689	139,4533	147,1544	148,1422	134,4276
Standard Deviation	56,30455	59,91567	64,41443	51,80333	53,74118	53,09148	39,82785	52,52444	59,43016	54,53196	59,79032	70,76421	69,76937	65,75944	72,31448	60,70475	55,9349	68,9867	62,9632	67,31027
Pixels	44055	37880	32211	34997	30295	23759	24356	41131	44976	50275	51224	26751	32411	46020	36881	39182	53350	17381	47348	28447
Area [µm x µm]	1898,06	1632,02	1387,77	1507,8	1305,22	1023,63	1049,35	1772,08	1937,74	2166,04	2206,93	1152,54	1396,39	1982,72	1588,97	1688,11	2298,52	748,84	2039,93	1225,61

	ROCK_UT Ch2	ROCK_UT Ch2	ROCK_UT Ch2	ROCK_UT Ch2	ROCK_UT Ch2	ROCK_UT Ch2	ROCK_UT Ch2	ROCK_UT Ch2	ROCK_UT Ch2	ROCK_UT Ch2	ROCK_LUC Ch2	ROCK_LUC Ch2	ROCK_LUC Ch2	ROCK_LUC Ch2	ROCK_LUC Ch2	ROCK_LUC Ch2	ROCK_LUC Ch2	ROCK_LUC Ch2	ROCK_LUC Ch2	ROCK_LUC Ch2
Mean Intensity	135,82361	120,9226	93,44732	117,5793	125,5272	126,5658	115,3254	113,378	138,4365	121,246	133,538	137,3856	127,3859	108,6287	130,2749	124,1738	129,9247	110,4445	119,7723	146,0645
Standard Deviation	54,55493	65,47503	43,58864	53,28856	55,24429	61,48045	55,18535	49,63098	53,07616	50,97395	64,2064	62,88833	68,44992	65,07061	61,24418	67,80456	63,18776	62,79474	67,07351	67,06709
Pixels	63497	27766	45086	50694	50351	44081	59834	53736	56544	50106	48787	40527	31040	33305	47277	21907	44217	41486	29676	22920
Area [µm x µm]	2735,69	1196,27	1942,48	2184,09	2169,31	1899,18	2577,88	2315,15	2436,13	2158,76	2101,93	1746,06	1337,32	1434,91	2036,87	943,84	1905,04	1787,38	1278,56	987,48

	IMP Ch1	IMP Ch1	IMP Ch1	IMP Ch1	IMP Ch1	IMP Ch1	IMP Ch1	IMP Ch1	IMP Ch1	IMP Ch1
Mean Intensity	55,4455	46,74027	70,12173	57,70322	47,38352	39,49138	38,63119	45,76703	64,07427	67,43186
Standard Deviation	31,84122	21,25295	47,3552	37,24797	33,14255	30,91475	25,32009	31,35747	50,99733	35,90137
Pixels	27109	21087	46511	40037	34564	23267	28326	27437	33945	32471
Area [µm x µm]	1167,96	908,51	2003,87	1724,95	1489,15	1002,43	1220,39	1182,09	1462,48	1398,98

	ROCK_IMP Ch1	ROCK_IMP Ch1	ROCK_IMP Ch1	ROCK_IMP Ch1	ROCK_IMP Ch1	ROCK_IMP Ch1	ROCK_IMP Ch1	ROCK_IMP Ch1	ROCK_IMP Ch1	ROCK_IMP Ch1
Mean Intensity	36,70761	30,49593	46,08635	49,46251	41,21713	48,41071	74,51217	41,85829	66,29692	52,02578
Standard Deviation	19,72452	24,0557	36,46125	30,7247	24,93333	30,68228	39,15535	22,01753	39,33921	24,84614
Pixels	17323	19414	33978	33820	19620	37769	44052	25587	36138	9582
Area [µm x µm]	746,34	836,43	1463,9	1457,1	845,3	1627,23	1897,93	1102,39	1556,96	412,83

	IMP Ch2	IMP Ch2	IMP Ch2	IMP Ch2	IMP Ch2	IMP Ch2	IMP Ch2	IMP Ch2	IMP Ch2	IMP Ch2
Mean Intensity	20,58672	27,41252	35,65526	31,07808	35,84593	38,88906	21,33689	24,65969	18,87174	37,89808
Standard Deviation	10,64966	15,50517	17,94612	21,17804	29,44661	30,15602	14,04251	24,27526	14,50424	23,34994
Pixels	5979	14089	5964	17585	13046	9933	7691	16635	13706	9370
Area [µm x µm]	257,6	607,01	256,95	757,63	562,07	427,95	331,36	716,7	590,51	403,7

	ROCK_IMP Ch2	ROCK_IMP Ch2	ROCK_IMP Ch2	ROCK_IMP Ch2	ROCK_IMP Ch2	ROCK_IMP Ch2	ROCK_IMP Ch2	ROCK_IMP Ch2	ROCK_IMP Ch2	ROCK_IMP Ch2
Mean Intensity	43,95908	23,70724	24,91652	40,35716	32,1558	45,05644	61,40625	50,31695	71,20432	46,72421
Standard Deviation	26,87515	18,52554	20,09551	22,74094	14,57491	31,6362	28,04472	32,74511	40,98047	21,28654
Pixels	7184	7040	7954	7736	4217	7849	11365	10475	14032	2520
Area [µm x µm]	309,51	303,31	342,69	333,3	181,68	338,17	489,65	451,3	604,55	108,57

B

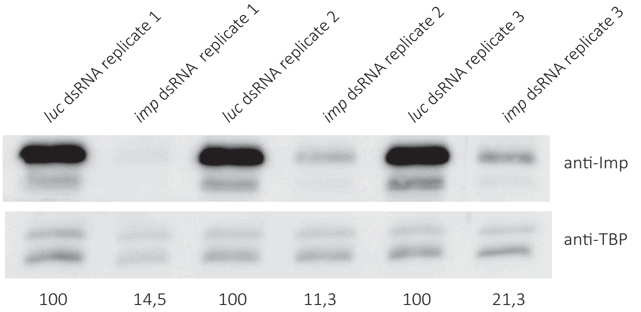

C

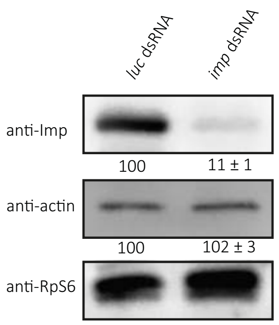

S4 Figure legend

(A) Table showing the mean intensity of Alexa Fluor 488 (green) and Alexa Fluor 568 (red) pixels (phalloidin and Imp staining, respectively). The pixels in ten visual fields from each dish (UT, dsRNA *imp*, dsRNA *luciferase*, UT + ROCK inhibitor, dsRNA *imp* + ROCK inhibitor, and dsRNA *luciferase* + ROCK inhibitor) were quantified using the HISTO functionality of the ZEN 2011 software package.

(B) Knockdown efficiency of Imp in S2 cells treated with *imp* dsRNA or *luciferase* dsRNA
S2 cells were treated with *imp* dsRNA or *luciferase* dsRNA. Western blot analysis of whole cell lysates from *imp* dsRNA- treated cells showed about 85% knockdown of Imp. The level of Imp was normalized to the TATA-binding protein (TBP) protein loading control.

(C) Western blot of *luc* dsRNA and *imp* dsRNA treated S2 cell lysate. Anti-Imp polyclonal antibody was used to detect the Imp protein. Anti-actin monoclonal antibody was used to detect the actin protein, while anti-Rps6 mouse monoclonal antibody was used to detect the presence of RpS6. RpS6 was used as a loading control and quantification of the intensities was performed in triplicates using ImageJ.

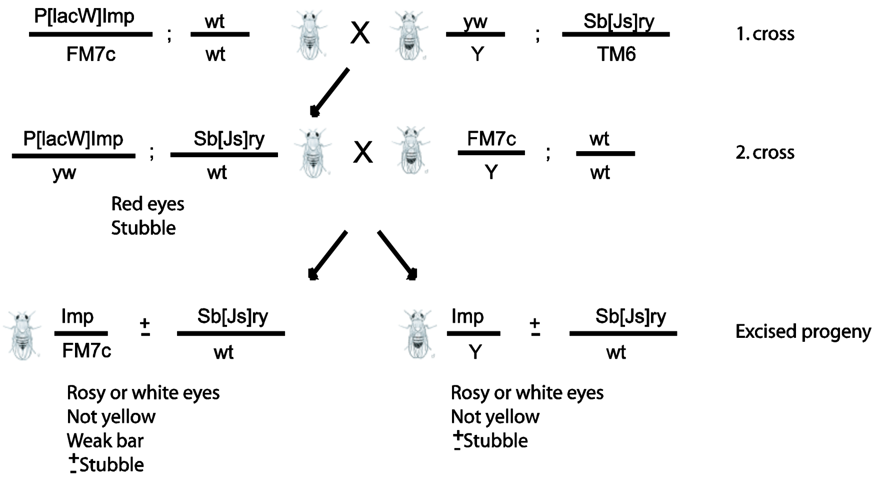

S5 Figure legend

Outline of the crosses that led to excision of the P-element.

Females are shown to the left. 1. cross: Imp^{G0072} heterozygous females were crossed to “jumpstarter” males that expressed a transposase that can induce the P-element to jump. Progeny possessing both the disrupted *imp* gene and the *transposase* gene was recognized by its phenotypic markers, red eyes from the P-element and stubble from the *transposase*. 2. cross: Female progeny from the first cross possessing a disrupted *imp* gene and a transposase was mated to FM7c balanced males. The transposase induced the P-element to jump, and progeny in which the P-element was excised could be recognized by their phenotypic markers, eye color and no bar from an FM7c allele.

Hansen_SupFig_S6

Protein	Putative function in neurons	References
	**Actin cytoskeleton dynamics**	
R (Rap1)	A small GTPase-like protein, that has been found to stimulate F-actin polymerization upon chemoattractant stimulation in *Dictyostelium*[2][2]. Rap1 controls inside-out signaling to integrins, with a model proposing that Rap1 contributes to the anchoring of integrins to the actin cytoskeleton[3][3]. Rap1 localizes to spiny and aspiny neurons in cultured rat neurons.	[2]; [3]; [4]
RhoGDI	Rho-GDP disassociation inhibitor. Removes Rho family GTPases from membranes and solubilizes them in the cytosol. It also inhibits GTP hydrolyzing activities of Rho-proteins.	[5]
Rin (Rasputin)	Rasputin encodes the RasGAP binding protein homolog G3BP, and has been shown to function in Ras- and Rho-mediated signaling in *Drosophila*.	[6]
Cdc42	In primary *Drosophila* embryo cultures, this Rho GTPase is responsible for inducing large growth cones and long filopodia by promoting the polymerization of actin.	[7, 8]
Rac1	In primary *Drosophila* embryo cultures, this Rho GTPase is responsible for inducing thick actin bundles. In mammalian cells, it is responsible for actin polymerization and the creation of lamellipodia.	[7, 8]
Moe (Moesin)	The sole *Drosophila* ERM protein, which is highly enriched in axons and other membrane protrusions, and promotes cortical actin assembly linking the membrane to the cytoskeleton.	[9]; [10]
Chic (Chickadee)	Encodes the G-actin binding protein profilin, and *chic* mutant γ-neurons fail to extend their axons.	[11]
Zip (Zipper)	Encodes the non-muscle myosin (myosin II) heavy chain, which is necessary for correct axon patterning and retrograde flow of the actin network in *Aplysia californica* bag cell neurons	[12]; [13]
Pp1-87B	Protein phosphatase-1. Influences cytoskeleton dynamics in the growth cone of photoreceptor cells in *Drosophila.*	[14]
Mys (Myospheroid)	Encodes an integrin-beta subunit. Integrins connect the extracellular matrix to the actin cytoskeleton. Is expressed in the CNS of *Drosophila* embryos, and loss of function mutants show axon guidance defects.	[15]
Msn (Misshapen)	A Ste20-like Serine/Threonine Kinase present in *Drosophila* photoreceptor axons and growth cones. Is involved in the reorganization of actin cytoskeleton to control growth cone motility.	[16]
	**Microtubule dynamics**	
Mapmodulin	Stabilizing proteins important for microtubule dynamics. Interacts with MAP1A in mammalian cells resulting in axonal and dendritic budding. Differentially regulated in *Drosophila* mushroom bodies γ-neurons at the onset of axonal pruning.	[17]; [18]
Ran	A small Ras family GTPase that localizes to CNS during *Drosophila* development. In *Drosophila* primary neurons and mouse neurons, knockdown of Ran caused increased branch arborization, indicating a role for Ran in regulating neurite extension. Furthermore Ran is a regulator of microtubule dynamics.	[19]; [20]
Sep1	Forms septin filaments in *Drosophila*, which are able to bind to microtubules *in vitro*. Some mammalian septins are localized to the tip of neurites where they are critical for neurite branching.	[21], [22]
Pnut (Peanut)	Forms septin filaments in *Drosophila*, which are able to bind to microtubules *in vitro*. Some mammalian Septins are localized to the tip of neurites where they are critical for neurite branching.	[21], [22]
	**Vesicle dynamics**	
Vsg (Visgun)	Locates to endocytotic compartments and may contribute to endolysosomal biogenesis and trafficking. Has been shown to regulate cell proliferation in a *Drosophila* cell line.	[23]
Vha16-1, Vha26, Vha68-2, Vha55	Subunits of vacuolar ATPase proton pumps that localize to subcellular vesicles. V-ATPases are also present in the membrane of synaptic vesicles in the neurons of *T. marmorata* fish.	[24]; [25]
SesB	Mitochondrial ATP/ADP translocase activity is essential for neurotransmission, and loss of SesB is accompanied by a complete loss of synaptic transmission in the visual system in *Drosophila*.	[26]
Surf4	A cargo receptor involved in intracellular protein trafficking. In rat PC12 cells, it localizes to neurites of neuronal cells and is associated with proteins that induce neurite formation by controlling directional membrane trafficking.	[27]
Sar1	Initiates formation of coated vesicles for forward trafficking from ER to Golgi. A genetic screen in *Drosophila* larval class IV dendritic arborization neurons showed defects in Sar1 expression resulted in decreased length of dendrites, due to dispersion of Golgi outposts in dendrites.	[28]
Rab11	Rab11 endosomes regulate the transport of beta-integrins to the axonal growth cone. In rat PC12 neuronal cells, Rab11 is needed for normal neurite outgrowth.	[29]
Rab5	Loss of the Rab5 endosomes result in a reduced number of dendritic branches in *Drosophila* dendritic arborization neurons, likely due to an inability to move proteins important for branching events along the microtubules.	[30]
Arf51F	The mammalian homolog ARF6 transports integrins along the axon as well as in the growth cone and enhance neurite outgrowth in rat dorsal root ganglion axons.	[31]
	**Membrane dynamics and signaling**	
Sty (Sprouty)	Sprouty is an antagonist of the EGFR and FGFR signaling pathway and co-localizes with membrane ruffles and prevents the production of excessive number of branching events in neurons and glia cells in *Drosophila*. Mislocalization of Spry causes incorrect distribution of branching event.	[32]; [33]
Arf79f	A GTPase involved in the recruitment of factors responsible for polarization of actin filaments needed in the	[34] 2012)

	ruffles and prevents the production of excessive number of branching events in neurons and glia cells in *Drosophila*. Mislocalization of Spry causes incorrect distribution of branching event.	
Arf79f	A GTPase involved in the recruitment of factors responsible for polarization of actin filaments needed in the formation of lamellipodia in *Drosophila* neurons.	[34] 2012)
Prominin-like	A transmembrane protein that localizes in membrane protrusions. Knockdown of Prominin-like resulted in a disruption of primary neuron formation in *Drosophila*.	[35]; [20]
14-3-3 epsilon	Is highly expressed in the embryonic brain, CNS and motor axons in *Drosophila*. Binds to PKC and Raf, protein kinases with critical roles in neuronal signaling.	[36]
Fax	Localizes to cellular membranes of CNS and PNS of *Drosophila* embryos. Deletions of Fax together with Abl (a tyrosin kinase that binds directly to F-actin) result in a disruption of the axonal architecture.	[37]
Eff (Effete)	An E2 ubiquitin-conjugated enzyme. A key regulator of ubiquitin-proteasome mediated pruning of mushroom body γ-neurons and class IV dendritic arborization sensory neurons during metamorphosis in *Drosophila*.	[38]; [39]
PRL-1	Tyrosine phosphatase, *Drosophila* PRL is mainly enriched in developing CNS during embryogenesis.	[40]
alpha-Spec	Spectrins (α and β) link the cell adhesion proteins in membranes to the F-actin cytoskeleton. Mutations have been shown to result in altered axon growth cone morphogenesis in *Drosophila* embryos.	[41]
Vap-33-1	In *Drosophila,* Vap is needed for axonal localization of the cell surface receptor Dscam, which is important for axon guidance.	[42]
Cnx99A	Transmembrane protein, containing two Ca^{2+} binding sites, that resides in ER and is necessary for correct function of the photoreceptor neuron in *Drosophila*.	[43]
	**Translational regulation**	
Hrb27c	Member of the hnRNP A/B family and binds to and regulates translation of *oskar* mRNA in *Drosophila*. Mutation of the *Hrb27c* gene caused a disruption of axonal projections in photoreceptor neurons in *Drosophila.*	[44]; [45]
pAbp	Cytoplasmic poly(A) binding protein, regulator of translational initiation.	[46][46]
Sqd (Squid)	An hnRNP required for correct localization and translational regulation of *gurken* mRNA. Sqd interacts with Hrb27c and an RNAi screen conducted in *Drosophila* larval class IV dendritic arborization neurons showed that knockdown of Squid resulted in both branch loss and decreased length of dendrites.	[47]; [48]
eIF-1A	Knockdown of translational initiation factor eIF-1A leads to severe defects in dendrite morphogenesis, resulting in characteristic trees with truncated main branches and few and short higher order branches.	[49]

S6 Figure legend

A subset of the 86 transcripts and their putative functions in neurons

A literature search revealed that 40 out of the 86 transcripts, identified as key Imp targets by a combination of RIP-seq, iCLIP and PAR-iCLIP, can be associated with neuronal development, with a predominant involvement in axonal growth cone development or guidance. The protein names, as well as a short summary of their involvement in neuronal development, are listed in column 1 and 2, respectively, and references are listed in column 3. The proteins are divided into functional boxes based on their referenced functions: “Actin cytoskeleton dynamics”, “Microtubule dynamics”, “Vesicle dynamics”, “Membrane dynamics and signaling”, and “Translational regulation”.

Supplemental Materials and methods

Immunostaining of S2 cells

Glass bottom dishes (GWSt-3522, Willco Wells) were treated with 40 µl 0.5 mg/ml concanavalin A (Sigma-Aldrich) [50]. 2x10^6 S2 cells were seeded in the petri-dish in 1 ml full medium and allowed to adhere to the bottom for 60 min. The medium was removed and cells were fixed by the addition of 1 ml fresh fixation solution [10% formaldehyde in PBS] and incubation for 10-15 min. Cells were washed briefly three times with PBS, and the cell membrane was permeabilized with 1 ml permeabilizing solution [PBS + 0.1% Triton X-100] for 3 min. Cells were washed briefly three times with PBS, and 500 µl blocking solution [1% normal goat serum (Sigma-Aldrich) in PBS] was added, followed by incubation for 1 h. 500 µl blocking solution containing a 1:10000 dilution of polyclonal antibody raised against Imp [51] was added, and the petri dish was incubated overnight at 4°C. Following three 5 min washes with PBS cells were incubated at room temperature for 1½ h with blocking solution containing 1:1000 Alexa Fluor® 568 goat anti-rabbit IgG (H+L)(Life Technologies). Three washes with PBS were completed in a dark container. To stain for cytoskeletal F-actin, cells were incubated with 1:1000 Alexa Fluor® 488 phalloidin (Life Technologies) for 5 min followed by a wash with PBS.

RNA immunoprecipitation sequencing (RIP-seq)

The following procedure was performed in duplicates on separate days. The library preparation protocol was previously described by [52]. Primers and a schematic outline of the library preparation are seen in Figure SM1A and B, respectively.

For the immunoprecipitation sample and the input RNA-seq sample, 4x10^7 S2 cells were pelleted and lyzed in 100 µl 50 mM Tris–HCl (pH 8.0), 100 mM NaCl, 1% NP40, 1.5 mM EDTA, complete EDTA-free protease inhibitor cocktail (Roche), 1 U/µl RiboLock (Thermo Scientific) lysis buffer. The lysates were cleared by centrifugation at 4°C and 22000 *g* for 20 min.

Total RNA was extracted for the input RNA-seq sample by adding Tri Reagent (Sigma-Aldrich) to the lysate according to the manufacturer’s specifications, and subsequently 40 µg of input RNA was subjected to poly(A) RNA enrichment by using Poly(A)Purist MAG kit (Ambion) according to the manufacturer’s specifications.

Immunoprecipitation was accomplished by first conjugating 40 µl polyclonal anti-Imp antibody (22053, made by BioGenes) to 400 µl Protein A Dynabeads (Life Technologies), according to the manufacturer’s specifications. Before immunoprecipitation, 900 µl lysis buffer was added, and the total 1 ml of lysate was mixed with the beads, and Imp-RNP complexes were immunoprecipitated by rotation for 2 h at 4°C. Beads were washed three times in lysis buffer, before Tri Reagent (Sigma-Aldrich) was added to the beads, and immunoprecipitated RNA was isolated according to the manufacturer’s specifications.

Input poly(A) RNAs and immunoprecipitated RNAs were fragmented to approximately 280 nt by heating at 95°C for 3½ min in the presence of 50 mM Tris-HCl (pH 8.0) and 5 mM $MgCl_2$. The fragmented RNA was precipitated, washed in 70% ethanol and resuspended in 11 µl Te [10 mM Tris (pH 8.0), 0.1 mM EDTA]. Size distribution was checked by running the fragmented RNA on a Bioanalyzer equipped with an RNA 6000 pico chip (Agilent).

For reverse transcription of the fragmented RNA samples, 1 µl RT_random_primer (100 pmol) and 1 µl 10 mM dNTP mix were added to 10 µl of the fragmented RNA, and the mixture was preheated for 5 min at 65°C before cooling. The RT mix [4.0 µl 5 x PrimeScript buffer (Takara), 0.5 µl RNAsin Plus (40 U/µl , Promega), 1.0 µl PrimeScript RT (200U) (Takara), 2.5 µl RNase free H_20] was added, and reverse transcription was performed with the following program; 25°C for 10 min, 42°C for 60 min, 70°C for 15 min and hold at 4°C.

The RNA was degraded by the addition of 0.75 µl RNAse H (5000 U/ml, New England Biolabs) and incubation for 20 min at 37°C. The cDNA was purified using RNAClean XP beads (Beckman Coulter) according to the manufacturer's specifications, and input cDNA was eluted from the beads using 20 µl H_2O, while the immunoprecipitated cDNA was eluted using 10 µl H_2O.

An adaptor was ligated to the 3'ends of the cDNAs by adding 7 µl ligation mix [1 µl CircLigase buffer (Epicentre), 0.5 µl 1 mM ATP, 0.5 µl 50 mM $MnCl_2$, 2 µl 50% PEG 6000, 2 µl 5 M betaine, 0.5 µl LIGATION_ADAPTER (100 µM), 0.5 µl CircLigase enzyme(Epicentre)] to 3 µl cDNAs. The mix was incubated as follows: 60°C for 2h, 68°C for 1h, 80°C for 10 min and put on hold at 4°C. The cDNAs were purified using AMPure XP beads (Beckman Coulter), according to the manufacturer's specifications.

Amplification of input and immunoprecipitated adaptor-ligated cDNA samples was performed by adding a PCR mix [3 µl PCR_forward 10 µM primer, 10 µl Phusion 5xHF buffer (Thermo Scientific), 1 µl dNTPs (10 mM), 24.5 µl H_2O and 1 µl Phusion® High-Fidelity DNA Polymerase (Thermo Scientific)] and 2.5 µl of indexing primer to 5 µl cDNA of each sample. Different indexing primers used for each sample is listed in Figure SMM1A.

The PCR_forward primer anneals to the 3' adaptor sequence while the PCR_reverse_index primer, containing the unique sample identifier sequence, anneals to the 5' adaptor sequence. The PCR was performed as follows; 98°C for 3 min; (98°C for 80 sec, 64°C for 15 sec, 72° C for 30 sec)x4 and (98°C for 80 sec, 72°C for 45 sec)x24 followed by 72°C for 5 min. The PCR product was purified using AMPure XP beads (Beckman Coulter) according to the manufacturer's specifications and resuspended in 11 µl H_2O.

The PCR samples were run in a 2% E-Gel SizeSelect agarose gel (Life Technologies) and the libraries of 210-500 bp were collected, purified using AMPure XP beads (Beckman Coulter) and resuspended in 11 µL H_2O. The size and the concentration of the libraries were examined on a Bioanalyzer equipped with a DNA 1000 chip (Agilent Technologies), and molar equivalents of the different libraries were pooled and subjected to Illumina sequencing.

Figure SMM1

A

**Oligonucleotide name**	**Sample**	**Sequence (5'to 3')**
RT_random_primer		AGACGTGTGCTCTTCCGATCTNNNNNNNNS
LIGATION_ADAPTER		PHO-AGATCGGAAGAGCGTCGTGTAGGGAAAGAGTGT-3NHC3
PCR_forward		AATGATACGGCGACCACCGAGATCTACACTCTTTCCCTACACGACGCT
PCR_REVERSE_INDEX.20_GTGGCC	RNAseq1 (Input)	CAAGCAGAAGACGGCATACGAGATGGCCACGTGACTGGAGTTCAGACGTGTGCTCTTCCGATCT
PCR_REVERSE_INDEX.21_GTTTCG	RNAseq2 (Input)	CAAGCAGAAGACGGCATACGAGATCGAAACGTGACTGGAGTTCAGACGTGTGCTCTTCCGATCT
PCR_REVERSE_INDEX.22_CGTACG	RIPseq1 (IP)	CAAGCAGAAGACGGCATACGAGATCGTACGGTGACTGGAGTTCAGACGTGTGCTCTTCCGATCT
PCR_REVERSE_INDEX.23_GAGTGG	RIPseq2 (IP)	CAAGCAGAAGACGGCATACGAGATCCACTCGTGACTGGAGTTCAGACGTGTGCTCTTCCGATCT

B

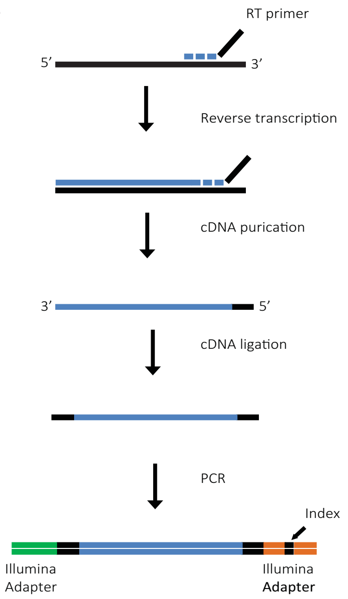

SMM1A: Oligonucleotides used in the preparation of the library [52]

SMM1B: A schematic outline of the library preparation.

iCLIP and PAR-iCLIP protocol

The methods used were adapted from [53] and [54]. Primers used in the preparation of the libraries and outlines of the procedures are depicted in Figures SMM2A and B, respectively.

For iCLIP and PAR-iCLIP, 5 x 10^7 S2 cells were plated in five 10 cm petri dishes, at a density of $2x10^5$ cells/ml and allowed to adhere to the bottom for 10 min. For PAR-iCLIP, cells were incubated with 100 µM 4-thiouridine (Sigma-Aldrich) for ~ 16 hours. Following a wash with ice-cold PBS, cells were subjected to UV-irradiation (250 mJ/cm^2 at 254 nm for iCLIP or 250 mJ/cm^2 at 365 nm for PAR-iCLIP) on ice. 1 ml ice-cold PBS was added and the cells were scraped off, transferred to 1.5 ml tubes and precipitated by centrifugation at 14,000 *g* for 2 min. Cells were snap-frozen on dry ice and stored at -80°C.

Cell pellets were resuspended in 1 ml lysis buffer [50 mM Tris–HCl (pH 8.0), 100 mM NaCl, 1% NP-40, complete EDTA-free protease inhibitor cocktail (Roche)]. For partial RNase digestion, the cross-linked lysate was incubated with RNase T1 (0.005 U/µl for iCLIP and 0.05 U/µl for PAR-iCLIP) (Thermo Scientific) in the presence of 0.0025 U/µl DNase I (Thermo Scientific) for 10 min at 37°C and 1000 rpm. Cell lysates were cleared by centrifugation at 4°C and 22,000 *g* for 20 min.

Imp-RNA complexes were immunoprecipitated by mixing the supernatant with 150 µl Protein A-coated Dynabeads (Life Technologies), pretreated with 5 µl polyclonal anti-Imp antibody [51] according to the manufacturer's specifications. Immunoprecipitation was performed for 2 h at 4°C on a rotating wheel, and the beads were subsequently washed three times with high-salt buffer [50 mM Tris-HCl (pH 7.4), 500 mM NaCl, 1% NP-40, 1 mM EDTA, 0.125% SDS] and twice with wash buffer [20 mM Tris-HCl (pH 7.4), 10 mM $MgCl_2$, 0.5% NP-40].

The 3' ends of the RNA was dephosphorylated by adding 80 µl PNK mix [60 µl H_2O, 16 µl 5xPNK buffer (350 mM Tris-HCl, pH 6.5, 50 mM $MgCl_2$, 25 mM dithiothreitol), 2 µl T4 polynucleotide kinase (Thermo Scientific), and 2 µl RNAsin Plus (40 U/µl, Promega)], to the beads and incubating for 20 min at 37°C and 800 rpm. The beads were washed twice with high-salt buffer and twice with wash buffer.

A pre-adenylated linker was ligated to the 3' ends of the RNA by adding 80 µl ligation mix [36 µl water, 16 µl 4x ligation buffer (200 mM Tris-HCl, pH 7.5, 40 mM $MgCl_2$, 40 mM DTT), 4 µl T4 RNA ligase (10 U/µl, Thermo Scientific), 2 µl RNAsin Plus (40 U/µl, Promega), 6 µl pre-adenylated linker L3 (20 pM, Integrated DNA Technologies), 16 µl PEG400] to the beads and incubating overnight at 16°C. The beads were washed twice with high-salt buffer and twice with wash buffer.

The RNA 5' end labeling was performed by adding [γ-^{32}P]ATP and T4 PNK mix [8 µl 10xPNK buffer, 1 µl [γ-^{32}P]ATP, 3.8 µl T4 polynucleotide kinase (Thermo Scientific), 67.2 µl H_2O] to the beads and incubating at 37° for 15 min at 1000 rpm. Beads were washed twice with high-salt buffer and twice with wash buffer, before they were resuspended in 45 µl 1X Novex® Tris-glycine SDS sample buffer (Life Technologies) containing 100 mM DTT, and incubating at 70°C for 10 min at 1000 rpm.

The samples were loaded on a 10% Bis-Tris gel [350 mM Bis-Tris (pH 6.6), 10% acrylamide: bis-acrylamide 37.5:1(National Diagnostics)] along with a PageRuler pre-stained protein ladder (Pierce) and run at 150 V for 90 min in 1x NuPAGE MOPS SDS Running Buffer (Life Technologies). The gel was equilibrated for 5 min in transfer buffer [2 X NuPage transfer buffer (Life Technologies), 1/10 volume methanol], and the RNA-

protein complexes were transferred to a Protran nitrocellulose membrane (Sigma-Aldrich) using semi-dry transfer (BioRad) at 20 V for 3 h. The membrane was washed in PBS, wrapped in cling film and exposed to an X-ray film (AGFA) at -80°C.

The region of interest (76-200 kDa for iCLIP and 70-120 kDa for PAR-iCLIP) was cut from the membrane, and proteins were digested by incubating the membrane in 200 µl auto-digested PK buffer [100 mM Tris-HCl (pH 7.4), 50 mM NaCl, 10 mM EDTA] with 15 µl Proteinase K (Roche) for 30 min at 55°C and 1000 rpm. Incubation was repeated after addition of 200 µl PK-urea buffer [100 mM Tris-HCl (pH 7.4), 50 mM NaCl, 10 mM EDTA, 7 M urea]. Samples were cooled to 37°C, mixed with 400 µl acidic phenol/chloroform (pH 4.5, Sigma-Aldrich), moved to 2 ml phase lock gel heavy tubes (cat. no. 713-2536, VWR) and incubated 15 min at 37°C and 1000 rpm. After centrifugation for 5 min at 13,000 g the aqueous phase was mixed with 1 µl GlycoBlue (Ambion), 1:10 volume 3 M sodium acetate and 2.5 x volume 100% ethanol and incubated overnight at -80°C. The RNAs were precipitated by centrifugation for 15 min at 15,000 *g* and 4°C, washed twice with 75% ethanol and resuspended in 12 µl H_20.

For reverse transcription, 1 µl 0.5 µM Rclip primer and 1 µl 10 mM dNTP mix were added to the RNA, and the mix was preheated for 5 min at 70°C before cooling to 25°C. The RT mix [4 µl 5 x Superscript RT buffer, 1 µl 0.1 M DTT, 0.5 µl Superscript III reverse transcriptase (Life Technologies)] was added, and reverse transcription was performed with the following program: 5 min at 25°C, 20 min at 42°C, 40 min at 50°C, 5 min at 80°C and hold at 4°C. cDNAs were precipitated by addition of 80 µl Te [10 mM Tris (pH 8.0), 0.1mM EDTA], 0.5 µl Glycoblue (Ambion), 10 µl 3 M sodium acetate and 250 µl ethanol overnight at -20°C and centrifugation for 15 min at 15,000 *g* and 4°C. Pellets were washed in 80% ethanol and resuspended in 6 µl H_20.

For size separation, cDNAs were mixed with 6 µl urea load buffer [8 M urea, 20 mM Tris-HCl (pH 7.8), 1 mM EDTA, 0.04% xylene cyanol, 0.04% bromophenol blue] and samples were run in a 6% TBE-7 M urea gel [1X TBE [89 mM Tris, 89 mM H_3BO_3, 2.5 mM EDTA], 6% acrylamide/bis solution (19:1) (SERVA), 7 M urea] along with 5 µl ^{32}P-labelled (250 cps) ultra-low DNA marker (Thermo Scientific) in 1X TBE running buffer for 40 min at 13 W. The gel was exposed to X-ray film (AGFA) at 4°C for 30 min to 1 h. Two bands were cut from the gel corresponding to cDNA sizes of 100-170 nt and 170-300 nt. Gel fragments were mixed with 400 µl Te [10 mM Tris (pH 8.0) , 0.1 mM EDTA] and incubated at room temperature overnight. The solution was moved to a new tube, and cDNA was precipitated by adding 1:10 volume 3 M sodium acetate, 1 µl GlycoBlue (Ambion) and 2.5 x volume ethanol and incubating overnight at -20°C, followed by centrifugation at 20,000 *g* for 20 min at 4°C.

In order to circularize the cDNAs, the pellets were resuspended in 8 µl ligation mix [6,5 µl water, 0.8 µl 10x CircLigase Buffer II, 0.4 µl 50 mM $MnCl_2$, 0.3 µl Circligase II (Epicentre)] and incubated for 1 h at 60°C.

An oligodeoxynucleotide, complementary to the *Bam*HI restriction site in the Rclip primer, was annealed to the circular cDNA by adding 30 µl annealing mix [25 µl water, 4 µl 10x *Bam*HI Buffer (Thermo Scientific), 1 µl cut_oligo (10 µM)] and incubating for 1 min at 95°C followed by cooling to 25°C. The cDNA was linearized by adding 2 µl of a 10 U/µl *Bam*HI restriction enzyme (Thermo Scientific) and incubating for 60 min at 37°C. The cDNA was precipitated by adding 1:10 volume 3 M sodium acetate, 1 µl GlycoBlue (Ambion) and 2.5 x volume ethanol and incubating overnight at -20°C. The cDNA was pelleted by centrifugation for 20 min at 20,000 *g* and 4°C, and washed with 80% ethanol and resuspended in 20 µl H_2O.

For high-throughput sequencing, 10 µl of cDNAs were PCR-amplified by adding a PCR mix [0.5 µl primer mix P5/P3 Illumina (10 µM each), 10 µl Accuprime Supermix 1 (Life Technologies)] and incubating with the following program (94°C for 2 min, (94°C for 15 sec, 65°C for 30 sec, 68°C for 30 sec) x 21 (34 cycles for PAR-iCLIP replicate 1), 68°C for 3 min. The PCR products were purified using AMPure XP beads (Beckman Coulter) according to the manufacturer's specifications and resuspended in 11 µl H_2O.

The size and the concentration of the library were examined on a Bioanalyzer equipped with a DNA 1000 chip (Agilent). Molar equivalents of different libraries were mixed, and the sample was run on a 2% SizeSelect E-Gel (Life Technologies) in order to remove the RT/Illumina primer peak of 75 nt. The final library was purified using AMPure XP beads (Beckman Coulter) according to the manufacturer's specifications and resuspended in 11 µl H_2O. The final library was sequenced on a llumina flow cell.

RNA-seq for normalizing iCLIP data

RNA-seq was performed on cells harvested on the same days as the cells used in the corresponding biological iCLIP replicates. For each replicate, total RNA was isolated from $5x10^7$ cells using the standard TRI Reagent protocol (Sigma-Aldrich). Poly(A) RNA was enriched from 100 µg total RNA using Poly(A)Purist MAG kit (Ambion) according to the manufacturer's specifications.

The poly(A) RNA was fragmented to approximately 280 nt by heating at 95°C for 3½ min in the presence of 50 mM Tris-HCl (pH 8.0) and 5 mM $MgCl_2$. The poly(A) RNA was precipitated, washed in 70% ethanol and resuspended in 16 µL Te [10 mM Tris (pH 8.0), 0.1 mM EDTA]. Size distribution was examined on a Bioanalyser equipped with a RNA 6000 Pico chip (Agilent).

The 3' termini of 7.5 µl of poly(A) RNAs were dephosphorylated by adding PNK mix [2 µl 5x PNK buffer (350 mM Tris-HCl, pH 6.5, 50 mM $MgCl_2$, 25 mM DTT), 0.4 µl T4 polynucleotide kinase (Thermo Scientific), and 0.1 µl RNAsin Plus (40 U/µl, Promega)] and incubating for 20 min at 37°C. The enzyme was inactivated at 70°C for 10 min.

A pre-adenylated linker was ligated to the 3' ends of the poly(A) RNA by adding 5.9 µl ligation mix [0.5 µl 200 mM Tris (pH 8.8), 1 µl T4 RNA ligase (10 U/µl, Thermo Scientific), 0.2 µl RNAsin Plus (40 U/µl, Promega), 1.2 µl pre-adenylated linker L3 (20 µM), 3 µl PEG400]. The mixture was incubated overnight at 16°C followed by 20 min at 37°C. The enzyme was inactivated at 70°C for 10 min and 15 µl of H_2O was added.

The poly(A) RNAs with attached 3' linker were purified using RNAClean XP beads (Beckman Coulter) as recommended by the manufacturer. The RNA was eluted in 11 µl RNase-free water.

The remaining part of the library preparation followed the iCLIP procedure beginning with the reverse transcription of the RNAs. 17 cycles of PCR were used to amplify the cDNAs.

Figure SMM2

A

Oligonucleotide name	Sample ID	Sequence (5'to 3')
L3		/5rApp/AGATCGGAAGAGCGGTTCAG/3ddC
Cut_oligo		GTTCAGGATCCACGACGCTCTTCAAAA
P5Solexa		AATGATACGGCGACCACCGAGATCTACACTCTTTCCCTACACGACGCTCTTCCGATCT
P3Solexa		CAAGCAGAAGACGGCATACGAGATCGGTCTCGGCATTCCTGCTGAACCGCTCTTCCGATCT
Rclip1	iCLIP 1	X33NNAACCNNNAGATCGGAAGAGCGTCGTGGATCCTGAACCGC
Rclip12	iCLIP 2	X33NNGTGGNNNAGATCGGAAGAGCGTCGTGGATCCTGAACCGC
Rclip2	PAR-iCLIP 1	X33NNACAANNNAGATCGGAAGAGCGTCGTGGATCCTGAACCGC
Rclip5	PAR-iCLIP 2	X33NNCGCCNNNAGATCGGAAGAGCGTCGTGGATCCTGAACCGC
Rclip13	RNAseq 1	X33NNTCCGNNNAGATCGGAAGAGCGTCGTGGATCCTGAACCGC
Rclip16	RNAseq 2	X33NNTTAANNNAGATCGGAAGAGCGTCGTGGATCCTGAACCGC

B

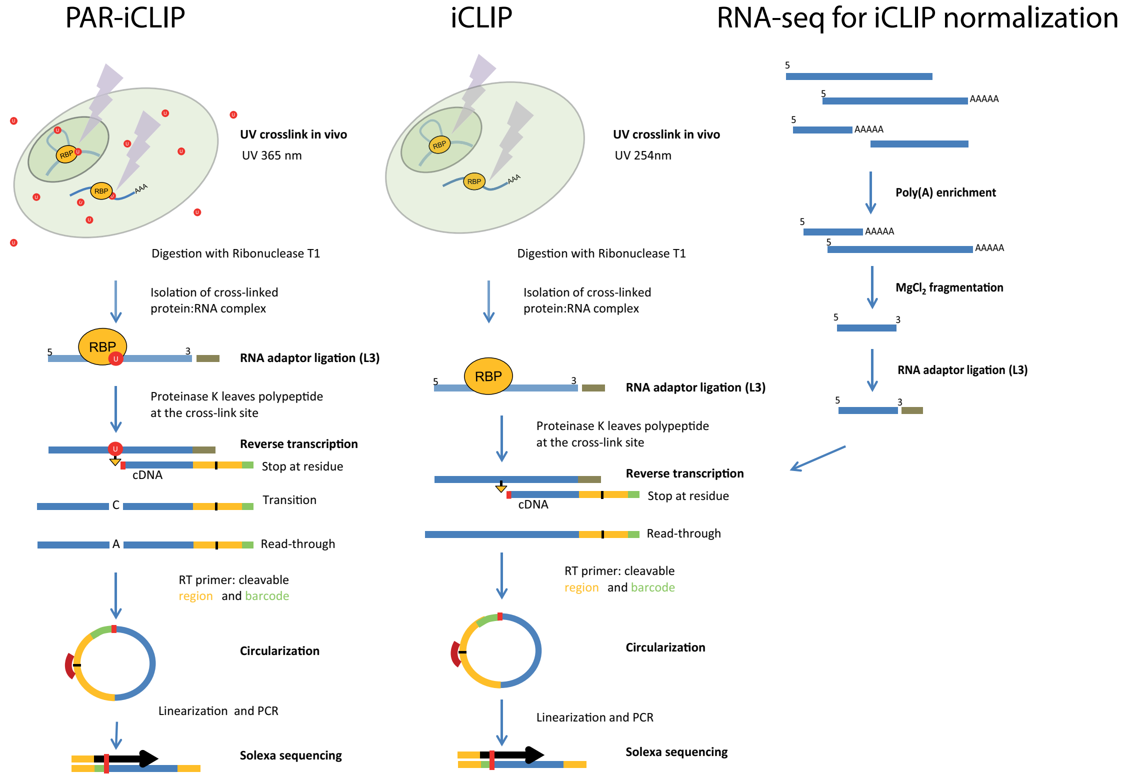

Figure adapted from [55]; [56]

Electrophoretic mobility-shift assay

DNA templates for *in vitro* transcription were synthesized from cDNAs with the primers: PABPCDST7-F + PABPCDS-R, PABP3UTRT7-F + PABP3UTR-R, SqdCDST7-F + SqdCDS-R and Sqd3UTRT7-F + Sqd3UTR-R. ^{32}P–labelled transcripts were generated by T7 RNA polymerase and purified by denaturing gel electrophoresis. The Imp coding region was amplified from EST SD7045 with the primers Imp-Bam-start and Imp-Xho-stop and inserted into the *Bam*HI and *Xho*I sites of pET28a (Novagen). Recombinant protein was expressed in RNase E-deficient *Escherichia coli* cells (Invitrogen) containing plasmid-encoded tRNAs for rare Arg, Ile and Leu codons and subsequently purified on Ni-agarose beads (Sigma-Aldrich). Radiolabelled RNA and 100 ng *Escherichia coli* tRNA were incubated with recombinant Imp at concentrations of 12.5 nM, 25 nM, 50 nM or 100 nM for 30 min at 30°C in 10 µl 20 mM Tris–HCl (pH 7.8), 140 mM KCl, 2 mM MgCl2 and 0.1% Triton X-100. After addition of Ficoll (2%) and bromophenol blue, samples were applied directly to a 1 mm 5% polyacrylamide gel (19:1) in 90 mM Tris-borate (pH 8.3), and run at 80 V for 3 h.

Figure SMM3

**Oligonucleotide name**	**Sequence (5'to 3')**
PABPCDST7-F	CTAATACGACTCACTATAGGGGTCGCTCCAAGGGATTCG
PABPCDS-R	TGTCCCAGCTGCTGCATACG
PABP3UTRT7-F	CTAATACGACTCACTATAGGGCATTCACTGATGCTTTGCGCA
PABP3UTR-R	GGACTGCAGAATTTGATTGCGTG
SqdCDST7-F	CTAATACGACTCACTATAGGGGTGGTCTGAGCTGGGAAAC
SqdCDS-R	CTTGGGATCGACCTTCTTGCTG
Sqd3UTRT7-F	CTAATACGACTCACTATAGGGTTGAAACACACAAACACCCACA
Sqd3UTR-R	AGACATTGAATCAAAGCTTTGATTACG
Imp-Bam-start	TAGCGGATCCATGCACAGCAACAATAATAGCA
Imp-Xho-stop	CGACAGGCTCGAGTT

Imp double-stranded RNA interference-mediated knockdown in S2 cells

The primers dsRNA Imp 1-F + dsRNA Imp 1&2-R and dsRNA Imp 2-F + dsRNA Imp 1&2-R, containing the T7 promoter sequence at the 5' end, were used to amplify a 343 and 479 bp fragment, respectively, of the Imp coding region using a pRmHa-4 Imp-FLAG plasmid as template. As a control dsRNA, primers dsRNA Luc 1-F + dsRNA Luc 1-R and dsRNA Luc 2-F + dsRNA Luc 2-R, containing the T7 promoter sequence at the 5' end, were used to amplify a 533 bp and 562 bp fragment, respectively, of the luciferase coding region using the pGL3 plasmid as template. Primer sequences are listed in Figure SMM4. The PCR product was purified using AMpure XP beads (Beckman Coulter) according to the manufacturer's specifications, and transcripts were generated by adding a reaction mix [10 µl 10x T7 RNA buffer (400 mM Tris-HCl, pH 7.5, 60 mM $MgCl_2$, 50 mM DTT, 10 mM spermidin (Sigma-Aldrich)), 10 µl 7 mM rNTPs (Amersham), 1.5 µl T7 RNA polymerase and up to 100 µl H_2O] to 2 µg of PCR template and incubating overnight at 37°C. The dsRNA was purified using AMpure XP beads (Beckman Coulter), and the concentration of the dsRNA was estimated by running an aliquot on a 1% agarose gel in the presence of an RNA dilution series of known concentration.

S2 cells were pelleted and resuspended in FBS- and antibiotics-free Schneider's *Drosophila* medium (Biowest) at a density of 1x10^6 cells/ml. One ml cell suspension was seeded in a 6-well tissue culture dish. Following adhesion, the medium was removed and 1 ml serum-free medium containing 40 µg/ml dsRNA Imp 1 or dsRNA Imp 2 was added to the well. The control cell culture was treated with 1 ml serum-free medium containing 40 µg/ml of dsRNA Luc 1 or dsRNA Luc 2. The dish was incubated at 26°C for 60 min, and 2 ml full medium (containing heat-inactivated FBS and antibiotics) was added to each well. Cells were incubated for 3 days to allow turnover of the targeted proteins. Each day, cells were spun down, medium removed, and a boost of serum-free medium containing 40 µg/ml dsRNA Imp 1/dsRNA Imp 2 or 40 µg/ml of dsRNA Luc 1/dsRNA Luc 2 was added.

Cells were washed twice in PBS, prior to addition of Tri Reagent (Sigma-Aldrich), and total RNA and protein extracts were isolated from the samples following the manufacturer's instructions, with the following modifications. Proteins were precipitated by adding 3 X acetone and incubating at RT for 10 min, followed by centrifugation at 12,000 *g* for 10 min at 4°C. Precipitated proteins were washed twice with protein wash 1 buffer [300 mM guanidine hydrochloride (Sigma-Aldrich) in 95% ethanol + 2.5% glycerol (v:v)] and twice with protein wash 2 buffer [ethanol containing 2.5% glycerol (v:v)]. The proteins were resuspended in 1% SDS with 100 mM DTT, and protein concentration was measured using the Qubit® Protein Assay Kit (Life Technologies) according to the manufacturer's specifications.

To test for efficient knockdown of Imp, western blot was performed with a SDS-PAGE electrophosesis system. Briefly, 2 µg total protein sample was resuspended in SDS-load buffer [50 mM Tris-HCl (pH 6.8), 100 mM DTT, 0.05% bromophenol blue, 10% glycerol] and electrophoresed in a 10% SDS-PAGE gel with SDS-running buffer [25 mM Tris, 192 mM glycine, 0.1% SDS]. The proteins were blotted to a PVDF membrane (Amersham Hybond-P), and the upper half was probed with polyclonal anti-Imp antibody (Adolph, 2009), whereas the lower half was probed with monoclonal anti-TBP antibody (SC Biotechnology). Horseradish peroxidase-conjugated anti-rabbit IgG (Cell Signaling) or anti-mouse IgG (Cell signaling) were then added, respectively, and the secondary antibodies were detected using enhanced chemiluminescence (Chemiluminescent Substrate from Chemiluminescent Nucleic Acid Detection Module, Thermo Scientific). Chemiluminescence was detected using the luminescent image analyzer LAS-1000plus, and ImageJ was used to quantify the Imp/TBP ratio.

Figure SMM4

**Oligonucleotide name**	**Sequence (5'to 3')**
dsRNA Imp 1-F	TAATACGACTCACTATAGGAAGTTATGCAGCAGGAGGC
dsRNA Imp 1&2-R	TAATACGACTCACTATAGGTGTCGACATCATTGCCATC
dsRNA Imp 2-F	TAATACGACTCACTATAGGACCATCAGGACGATCACACA
dsRNA Luc 1-F	TAATACGACTCACTATAGGAGAACTGCCTGCGTGAGATT
dsRNA Luc 1-R	TAATACGACTCACTATAGGCTTGGCATTCCGGTACTGTT
dsRNA Luc 2-F	TAATACGACTCACTATAGGCATCGACTGAAATCCCTGGT
dsRNA Luc 2-R	TAATACGACTCACTATAGGAGAGCAACTGCATAAGGC

Western Blot of actin protein levels in Imp deficient S2 cells

Knock-down of *imp* and western blot was performed as previously described. Following blotting of proteins to a PVDF membrane (Amersham Hybond-P), the upper half was probed with polyclonal anti-Imp antibody (Adolph, 2009), the middle part with polyclonal anti-actin antibody (Cytoskeleton) whereas the lower half was probed with monoclonal anti-S6 ribosomal protein antibody (Cell signaling). Horseradish peroxidase-conjugated anti-rabbit IgG (Cell Signaling) or anti-mouse IgG (Cell signaling) were then added, and the secondary antibodies were detected using enhanced chemiluminescence (Chemiluminescent Substrate from Chemiluminescent Nucleic Acid Detection Module, Thermo Scientific). Chemiluminescence was detected using an SLR camera (Khoury 2010) and ImageJ was used to quantification.

RNA-seq on Imp double-stranded RNA interference-mediated knockdown

The following experiment was carried out in biological triplicates on different days. Primers used for the library preparation are listed in Figure SMM5.

dsRNA treatment with dsRNA Imp 1/dsRNA Imp 2 and dsRNA Luc1 were performed on $5x10^6$ cells, as previously described. Total RNA and protein extracts were isolated from the TRI Reagent (Sigma-Aldrich) samples following the manufacturer's instructions. Total RNA concentration was measured using NanoDrop, and poly(A) RNA was enriched from 30 µg total RNA using the Poly(A)Purist MAG kit (Life Technologies) according to the manufacturer's specifications. The poly(A) RNA was fragmented to approximately 280 nt by heating at 95°C for 3.5 min in the presence of 50 mM Tris-HCl (pH 8.0) and 5 mM $MgCl_2$. The poly(A) RNA was precipitated, washed in 70% ethanol and resuspended in 11 µl Te [10 mM Tris (pH 8.0), 0.1 mM EDTA]. Size distribution was examined on a Bioanalyzer equipped with a RNA 6000 Pico chip.

The conversion of poly(A) RNA into cDNA containing 5' and 3' adaptors ready for PCR amplification was performed as previously described (RIP/RNAseq section) with the exception that the 3' ligation adaptor (LIGATION_ADAPTER_RB) in these experiments contain a 7-mer random barcode, making it possible to collapse PCR duplicates.

PCR was performed using the Phusion DNA polymerase (Thermo Scientific) as previously described (RIP-seq section) with 20 cycles of amplification, creating libraries with the index primers indicated in Figure SMM5. E-gel size selection and AMPure XP beads (Beckman Coulter) purification were performed as previously described (RIP-seq section). Molar equivalents, as assessed by a Bioanalyzer equipped with a DNA 1000 chip, were mixed of all the final libraries, and the mixture was subjected to Illumina flow cell sequencing.

Figure SMM5

**Oligonucleotide name**	**Sample**	**Sequence (5'to 3')**
RT_random_primer		AGACGTGTGCTCTTCCGATCTNNNNNNNNS
LIGATION_ADAPTER_RB		PHO-NNNNNNNAGATCGGAAGAGCGTCGTGTAGGGAAAGAGTGT-3NHC3
PCR_forward		AATGATACGGCGACCACCGAGATCTACACTCTTTCCCTACACGACGCT
PCR_REVERSE_INDEX.2_CGATGT	Luc dsRNA treated 1	CAAGCAGAAGACGGCATACGAGATACATCGGTGACTGGAGTTCAGACGTGTGCTCTTCCGATCT
PCR_REVERSE_INDEX.4_TGACCA	Imp dsRNA treated 1	CAAGCAGAAGACGGCATACGAGATTGGTCAGTGACTGGAGTTCAGACGTGTGCTCTTCCGATCT
PCR_REVERSE_INDEX.5_ACAGTG	Luc dsRNA treated 2	CAAGCAGAAGACGGCATACGAGATCACTGTGTGACTGGAGTTCAGACGTGTGCTCTTCCGATCT
PCR_REVERSE_INDEX.6_GCCAAT	Imp dsRNA treated 2	CAAGCAGAAGACGGCATACGAGATATTGGCGTGACTGGAGTTCAGACGTGTGCTCTTCCGATCT
PCR_REVERSE_INDEX.7_ CAGATC	Luc dsRNA treated 3	CAAGCAGAAGACGGCATACGAGATGATCTGGTGACTGGAGTTCAGACGTGTGCTCTTCCGATCT
PCR_REVERSE_INDEX.24_GGTAGC	Imp dsRNA treated 3	CAAGCAGAAGACGGCATACGAGATGCTACCGTGACTGGAGTTCAGACGTGTGCTCTTCCGATCT

Embryo work

Imp^{G0072} strain

imp^{G0072} is obtained from Bloomington *Drosophila* Stock Center (stock number 11798, w67c23 P{lacW}ImpG0072/FM7c). As the *imp* gene is situated on the X-chromosome and the *imp^{G0072}* allele is kept as a balanced heterozygote, the only flies in this stock without a wild-type *imp* locus were the hemizygous males. To obtain a *imp^{G0072}* line in which the balancer expressing a wild-type *imp* allele could be marked, an FM7c balancer with a β-galactosidase gene inserted under control of an actin 5C promotor (FM7c, P{act-lacZ.B}GD1 P{act-lacZ.B}GD2, stock 2179 from Bloomington *Drosophila* Stock Center) was crossed into the *imp^{G0072}* line. Staining the resulting line with antibodies against β-galactosidase identified embryos with a balancer chromosome and thus at least one wt *imp* gene.

RT-qPCR on pharate adult mutants

Hemizygous *imp^{G0072}* mutants or wild-type pharate adult males were removed from their pupae under an Olympus SZ40 microscope and approximately 20 males were pooled. Males were recognized by their sex combs and hemizygous *imp^{G0072}* mutants were recognized by their red eye-colour. RNA was extracted from the pharate adults using Trizol reagent (Invitrogen) according to the instructions of the manufacturer, including the optional centrifugation step. The quantification was therefore carried out on a collection of pupae and did not reveal whether there were fluctuations in the levels between individual flies. cDNA was synthesized from total RNA isolated from wild-type or hemizygous *imp^{G0072}* pharate adult males with Superscript III (Invitrogen) using an oligo(dT) primer. Quantitative RT-PCR was performed with the LightCycler-FastStart DNA Master SYBR Green I kit (Roche) according to the instructions of the manufacturer. The following primers were used: Imp-F: GGTTGAGTTGCTTGTGGCCA, Imp-R: CGGCGGATTTGTCGAAAGCA, RP49-F: ATCCGCCCAGCATACAGGCCC and RP49-R: GTTCTCTTGAGAACGCAGGCGA. The relative amount of *imp* mRNA was normalized to the level of *rp49* mRNA in each sample.

Excision of P-element

In order to excise the P-element from the *imp* locus, *imp^{G0072}* females were crossed to jumpstarter males (y1 w*; ry506 Sb1 P{Δ2-3}99B/TM6, stock 3664 from Bloomington *Drosophila* Stock Center). Among F1 progeny, females with red eyes and a stubble phenotype, possessing a disrupted *imp* allele and a *transposase* gene were collected and crossed to FM7c/Y males. Among the F2 progeny, 53 flies in which the P-element had been excised were recognized by their eye color (white if not containing the *transposase* gene or rosy if containing the *transposase* gene) (Figure S5). 23 lines with the excised alleles were created by crossing excised females to FM7c/Y males. Genome DNA was extracted from males of these stocks and the region of the *imp* locus spanning the P-element insertion site was sequenced (with the primer Imp13208-F TGATTAACAAGTGAGCGCGT) to ensure that excision had taken place. DNA was also extracted from two flies that died a few days after eclosion, and the Imp genome region was sequenced. The sequence showed that both flies lacked part of the *imp* gene as a result of imprecise P-element excision.

Survival count

The fraction of *imp^{G0072}*, wild-type or FM7c/wt progeny able to proceed through embryogenesis, larval

stages and pupation was recorded. Flies that only managed to get halfway out of their pupae were not regarded as eclosed, but their number and phenotype was noted as well as the number and phenotypes of flies drowned in the medium. Eclosing flies were recorded once every 24 hours until no more eclosure was taking place. The phenotype of eclosing flies was also recorded. The percentage of flies that were unable to proceed into the next developmental stage was calculated. Males and females that were hemi/homozygous for the FM7c balancer were unhealthy, so to ascertain that the observed lethality was not associated with the FM7c balancer, the survival of the imp^{G0072} progeny was compared to both wild-type progeny and FM7c/wt progeny. The binominal p-value of imp^{G0072} compared to either wild-type or wt/FM7c were calculated. All obtained p-values were $p < 0.001$.

Immunostainings of mutant embryo

Wild-type embryos were collected from agar-juice plates, dechorionized for two minutes in 2% sodium hypochlorit and fixed in 4% paraformaldehyde. They were stained with anti-β-galactosidase antibody (Rockland, 1:10000) and monoclonal anti-Futsch 22C10 (1:100 from Developmental Studies Hybridoma Center, University Iowa). Secondary antibodies were anti-rabbit Alexa Fluor® 488 or anti-mouse Alexa Fluor® 555 from Molecular Probes (1:1000). Fluorescent immunostainings were visualized in a confocal Zeiss LSM510 microscope. In order to record the fraction of embryos with an abnormal neuronal pattern, 0-20 hours old wild-type, wt/FM7c or imp^{G0072} embryos were dechorionized, fixed and incubated with monoclonal anti-Futsch 22C10 (1:2000) overnight at 4°C. Following incubation with horseradish peroxidase-conjugated anti-mouse-IgG (Cell signaling), the embryos were stained with Sigma Fast™ 3,3'-diaminobenzidine tablet set (Sigma-Aldrich, D-4168) according to the instructions of the manufacturer. The distribution of embryos with a normally appearing morphology and nervous system and embryos with an abnormal morphology or nervous system was recorded by Zeiss Stemi SR microscopy. The percentage of abnormal embryos was estimated and the binominal p-value of imp^{G0072} embryos compared to either wild-type or wt/FM7c embryos were calculated. Obtained p-values were < 0.001.

Data treatment and bioinformatics analyses

Read mapping and processing

The following mapping procedure applies to the PAR-iCLIP, iCLIP, RIP and RNA-seq controls. Reads were preprocessed with custom python scripts. First, we de-multiplexed the reads according to their fixed barcode allowing up to 1 mismatch. Next, we trimmed base calls with one of the 5 lowest quality scores from 3' ends and removed adapter sequences. Finally, we removed duplicates by collapsing all identical reads containing the same random barcode and trimmed the 5' end of reads to remove the random barcodes. After these steps, all reads longer than 17 nucleotides were further analyzed.
The dmel3 version of the *Drosophila melanogaster* genome and ensembl72 annotation [57] were used to build the indexes. Reads were mapped to the dmel3 genome and an exon junction database using BWA-PSSM [58]. To map PAR-iCLIP reads, we used a custom matrix for scoring mismatches assuming a 12.5% T>C conversion rate. For further analysis, only reads mapped with a posterior probability (PP) > 0.99, referred to as confidently mapped reads, were used. Finally confidently mapped reads overlapping by one or more nucleotides were clustered. To perform motif discovery these large clusters were further divided in smaller regions with uniform coverage.

The RNA-seq datasets obtained from the Imp knock-down experiments used for the differential expression analyses were analyzed independently as they contained yeast RNA spike-in. In these datasets, we mapped the reads using BWA-PSSM as described above but we used a new genome index that included both *Saccharomyces cerevisiae* (sacCer3) and *Drosophila* (dmel3) genomes.

The human HuR PAR-CLIP data used in Figure S2C was obtained from [1] (GEO accession number GSE29780). The data was processed as described above. Reads were mapped also as described above using an index built from hg19 version of the human genome and an exon junction index extracted from ensembl70 annotation using the custom matrix to score T>C conversions. The RNA-seq used to normalize HuR PAR-CLIP data was a pool of RNA-seq datasets from [59] and [60] (GSM714684; GSM714685; GSM940576). These data were mapped to the same indexes as the HuR dataset.

Annotation

For this project the ensembl72 annotation of the *Drosophila melanogaster* was used. In all downstream analyses, one representative transcript was selected for each gene. This transcript was always the longest protein-coding transcript annotated for the gene.

3'UTR length versus coverage correlation analyses

In order to investigate the relationship between 3'UTR length and CLIP enrichment we pooled iCLIP and PAR-CLIP datasets and normalized them. The iCLIP replicates were normalized to their corresponding RNA-seq datasets, whereas PAR-iCLIP replicates were normalized to combined RNA-seq iCLIP control replicates, normalization described by in the methods section (equation 1). For each transcript the sum of enrichment was normalized to the 3'UTR length and log2 transformed.

Differential expression analysis

For each transcript we counted the number of confidently mapped overlapping reads on the same strand. Only unambiguously assigned reads (i.e. that only overlap one gene) were considered. Differential expression was calculated using DESeq R package [61]. We estimated dispersion using pooled-CR method, which treats replicates as paired data. The rest of the parameters were default settings. To correct for multiple hypothesis testing, we used Benjamini-Hochberg adjusted *p*-values and considered genes differentially expressed with a corrected *p*-value (FDR) < 0.05.

Co-occurrence analysis

To explore over-representation of repeated CA-rich motif occurrences we selected the 3000 most enriched CLIP clusters, and for each of these we extracted the sequence around cluster starts, beginning 40 nucleotides upstream from the cluster start and ending 50 nucleotides downstream. In these clusters we counted the number of co-occurrences of MACA (M is IUPAC nomenclature for A or C) separated by a particular distance. This count was then normalized by the number of possible words of that particular length as shown in equation 2.

$$f_i = \frac{cc_{wi}}{n(l_c-(2l_w+s_i)+1)} \qquad (2)$$

where n is the number of sequences, cc_w is co-occurrence count at a particular distance, l_w is the word length and s_i is the spacing and f is the normalized word frequency at the i'th spacing. As a control for the co-occurrence analysis we used a set of 3000 clusters 90 nt long randomly sampled from 3'UTRs of the longest protein-coding transcripts associated with genes.

Positional enrichment analysis

Using the same clusters defined above, we counted the occurrences of all 4-mers in each position of the 90 nt long clusters. Next, for each word we calculated their normalized frequency by dividing the word count in position c_{wi} by the number of sequences n as defined in equation 3

$$pf_i = \frac{c_{wi}}{n} \qquad (3)$$

This positional frequency was calculated independently for the iCLIP clusters pfC_i and for the background sequences $pfbg_i$. In these clusters, we defined the cross-link region as position 40 to 57 in the cluster, and flanking regions as the regions upstream and downstream of the cross-link region. Figures 2E and S2H show a running mean over 5 nucleotides of the positional frequencies described above. A mean Z-score Z_w for each word in the cross-link and flanking regions was calculated as described in equation 4. These Z-scores were plotted in the scatter plots in Figure 2D and Supplementary Figure S2G.

$$Z_w = \frac{\mu(pfC_i)-\mu(pfbg_i)}{\sigma(pfbg_i)} \qquad (4)$$

Standardized transcript profiles

To make standardized profiles, the longest protein-coding transcript of each gene with at least 30% RNA-seq coverage in S2 cells was used. For these transcripts, each region (5'UTR, CDS, 3'UTR) was divided in a

fixed number of equally sized bins as follows: 20 bins in 5'UTRs, 50 bins in CDS, and 50 bins in 3'UTRs. For iCLIP profiles, we calculated the mean enrichment of iCLIP reads relative to RNA-seq $\bar{e}_b$ for each bin *b* as

$$\bar{e}_b = \frac{1}{g}\sum_{i=1}^{g}\frac{1}{l_i}\sum_{j=1}^{l_i} e_i$$

where g is the total number of genes, l_i is the length of the bin which may differ by one if sequence length is not divisible by the number of bins. e_{ij} is the enrichment of iCLIP reads relative to RNA-seq calculated as described in the main text. For the standardized HMM profile, we calculated the match occurrence count for each bin b and inserted as the enrichment value e_{ij} in the equation above.

Supplemental References

1. Mukherjee N, Corcoran DL, Nusbaum JD, Reid DW, Georgiev S, Hafner M, Ascano M, Jr., Tuschl T, Ohler U, Keene JD: **Integrative regulatory mapping indicates that the RNA-binding protein HuR couples pre-mRNA processing and mRNA stability.** *Mol Cell* 2011, **43:**327-339.
2. Mun H, Jeon TJ: **Regulation of actin cytoskeleton by Rap1 binding to RacGEF1.** *Mol Cells* 2012, **34:**71-76.
3. Caron E: **Cellular functions of the Rap1 GTP-binding protein: a pattern emerges.** *J Cell Sci* 2003, **116:**435-440.
4. Fu Z, Lee SH, Simonetta A, Hansen J, Sheng M, Pak DT: **Differential roles of Rap1 and Rap2 small GTPases in neurite retraction and synapse elimination in hippocampal spiny neurons.** *J Neurochem* 2007, **100:**118-131.
5. Dovas A, Couchman JR: **RhoGDI: multiple functions in the regulation of Rho family GTPase activities.** *Biochem J* 2005, **390:**1-9.
6. Pazman C, Mayes CA, Fanto M, Haynes SR, Mlodzik M: **Rasputin, the Drosophila homologue of the RasGAP SH3 binding protein, functions in ras- and Rho-mediated signaling.** *Development* 2000, **127:**1715-1725.
7. Nobes CD, Hall A: **Rho, rac, and cdc42 GTPases regulate the assembly of multimolecular focal complexes associated with actin stress fibers, lamellipodia, and filopodia.** *Cell* 1995, **81:**53-62.
8. Matsuura R, Tanaka H, Go MJ: **Distinct functions of Rac1 and Cdc42 during axon guidance and growth cone morphogenesis in Drosophila.** *Eur J Neurosci* 2004, **19:**21-31.
9. Amieva MR, Furthmayr H: **Subcellular-Localization of Moesin in Dynamic Filopodia, Retraction Fibers, and Other Structures Involved in Substrate Exploration, Attachment, and Cell-Cell Contacts.** *Experimental Cell Research* 1995, **219:**180-196.
10. Edwards KA, Demsky M, Montague RA, Weymouth N, Kiehart DP: **GFP-moesin illuminates actin cytoskeleton dynamics in living tissue and demonstrates cell shape changes during morphogenesis in Drosophila.** *Developmental Biology* 1997, **191:**103-117.
11. Medioni C, Ramialison M, Ephrussi A, Besse F: **Imp promotes axonal remodeling by regulating profilin mRNA during brain development.** *Curr Biol* 2014, **24:**793-800.
12. Young PE, Richman AM, Ketchum AS, Kiehart DP: **Morphogenesis in Drosophila requires nonmuscle myosin heavy chain function.** *Genes Dev* 1993, **7:**29-41.
13. Lin CH, Espreafico EM, Mooseker MS, Forscher P: **Myosin drives retrograde F-actin flow in neuronal growth cones.** *Neuron* 1996, **16:**769-782.
14. Babu K, Bahri S, Alphey L, Chia W: **Bifocal and PP1 interaction regulates targeting of the R-cell growth cone in Drosophila.** *Dev Biol* 2005, **288:**372-386.
15. Hoang B, Chiba A: **Genetic analysis on the role of integrin during axon guidance in Drosophila.** *J Neurosci* 1998, **18:**7847-7855.
16. Ruan W, Long H, Vuong DH, Rao Y: **Bifocal is a downstream target of the Ste20-like serine/threonine kinase misshapen in regulating photoreceptor growth cone targeting in Drosophila.** *Neuron* 2002, **36:**831-842.
17. Opal P, Garcia JJ, Propst F, Matilla A, Orr HT, Zoghbi HY: **Mapmodulin/leucine-rich acidic nuclear protein binds the light chain of microtubule-associated protein 1B and modulates neuritogenesis.** *J Biol Chem* 2003, **278:**34691-34699.
18. Hoopfer ED, Penton A, Watts RJ, Luo L: **Genomic analysis of Drosophila neuronal remodeling: a role for the RNA-binding protein Boule as a negative regulator of axon pruning.** *J Neurosci* 2008, **28:**6092-6103.
19. Koizumi K, Stivers C, Brody T, Zangeneh S, Mozer B, Odenwald WF: **A search for Drosophila neural precursor genes identifies ran.** *Dev Genes Evol* 2001, **211:**67-75.

20. Sepp KJ, Hong P, Lizarraga SB, Liu JS, Mejia LA, Walsh CA, Perrimon N: **Identification of neural outgrowth genes using genome-wide RNAi.** *PLoS Genet* 2008, **4:**e1000111.
21. Sisson JC, Field C, Ventura R, Royou A, Sullivan W: **Lava lamp, a novel peripheral golgi protein, is required for Drosophila melanogaster cellularization.** *J Cell Biol* 2000, **151:**905-918.
22. Xie Y, Vessey JP, Konecna A, Dahm R, Macchi P, Kiebler MA: **The GTP-binding protein Septin 7 is critical for dendrite branching and dendritic-spine morphology.** *Curr Biol* 2007, **17:**1746-1751.
23. Zhou GQ, Zhang Y, Ferguson DJ, Chen S, Rasmuson-Lestander A, Campbell FC, Watt SM: **The Drosophila ortholog of the endolysosomal membrane protein, endolyn, regulates cell proliferation.** *J Cell Biochem* 2006, **99:**1380-1396.
24. Bohrmann J, Bonafede A: **Tissue-specific distribution and variation of the channel-forming protein ductin during development of Drosophila melanogaster.** *Int J Dev Biol* 2000, **44:**883-890.
25. Morel N, Dedieu JC, Philippe JM: **Specific sorting of the a1 isoform of the V-H+ATPase a subunit to nerve terminals where it associates with both synaptic vesicles and the presynaptic plasma membrane.** *J Cell Sci* 2003, **116:**4751-4762.
26. Rikhy R, Ramaswami M, Krishnan KS: **A temperature-sensitive allele of Drosophila sesB reveals acute functions for the mitochondrial adenine nucleotide translocase in synaptic transmission and dynamin regulation.** *Genetics* 2003, **165:**1243-1253.
27. Matsuzaki F, Shirane M, Matsumoto M, Nakayama KI: **Protrudin serves as an adaptor molecule that connects KIF5 and its cargoes in vesicular transport during process formation.** *Mol Biol Cell* 2011, **22:**4602-4620.
28. Ye B, Zhang Y, Song W, Younger SH, Jan LY, Jan YN: **Growing dendrites and axons differ in their reliance on the secretory pathway.** *Cell* 2007, **130:**717-729.
29. Eva R, Dassie E, Caswell PT, Dick G, ffrench-Constant C, Norman JC, Fawcett JW: **Rab11 and its effector Rab coupling protein contribute to the trafficking of beta 1 integrins during axon growth in adult dorsal root ganglion neurons and PC12 cells.** *J Neurosci* 2010, **30:**11654-11669.
30. Satoh D, Sato D, Tsuyama T, Saito M, Ohkura H, Rolls MM, Ishikawa F, Uemura T: **Spatial control of branching within dendritic arbors by dynein-dependent transport of Rab5-endosomes.** *Nat Cell Biol* 2008, **10:**1164-1171.
31. Eva R, Crisp S, Marland JR, Norman JC, Kanamarlapudi V, ffrench-Constant C, Fawcett JW: **ARF6 directs axon transport and traffic of integrins and regulates axon growth in adult DRG neurons.** *J Neurosci* 2012, **32:**10352-10364.
32. Kramer S, Okabe M, Hacohen N, Krasnow MA, Hiromi Y: **Sprouty: a common antagonist of FGF and EGF signaling pathways in Drosophila.** *Development* 1999, **126:**2515-2525.
33. Lim J, Wong ES, Ong SH, Yusoff P, Low BC, Guy GR: **Sprouty proteins are targeted to membrane ruffles upon growth factor receptor tyrosine kinase activation. Identification of a novel translocation domain.** *J Biol Chem* 2000, **275:**32837-32845.
34. Humphreys D, Liu T, Davidson AC, Hume PJ, Koronakis V: **The Drosophila Arf1 homologue Arf79F is essential for lamellipodium formation.** *J Cell Sci* 2012, **125:**5630-5635.
35. Nie J, Mahato S, Mustill W, Tipping C, Bhattacharya SS, Zelhof AC: **Cross species analysis of Prominin reveals a conserved cellular role in invertebrate and vertebrate photoreceptor cells.** *Dev Biol* 2012, **371:**312-320.
36. Skoulakis EM, Davis RL: **14-3-3 proteins in neuronal development and function.** *Mol Neurobiol* 1998, **16:**269-284.
37. Hill KK, Bedian V, Juang JL, Hoffmann FM: **Genetic interactions between the Drosophila Abelson (Abl) tyrosine kinase and failed axon connections (fax), a novel protein in axon bundles.** *Genetics* 1995, **141:**595-606.
38. Watts RJ, Hoopfer ED, Luo LQ: **Axon pruning during Drosphila metamorphosis: Evidence for local degeneration and requirement of the ubiquitin-proteasome system.** *Neuron* 2003, **38:**871-885.

39. Kuo CT, Zhu S, Younger S, Jan LY, Jan YN: **Identification of E2/E3 ubiquitinating enzymes and caspase activity regulating Drosophila sensory neuron dendrite pruning.** *Neuron* 2006, **51:**283-290.
40. Lin MD, Lee HT, Wang SC, Li HR, Hsien HL, Cheng KW, Chang YD, Huang ML, Yu JK, Chen YH: **Expression of phosphatase of regenerating liver family genes during embryogenesis: an evolutionary developmental analysis among Drosophila, amphioxus, and zebrafish.** *BMC Dev Biol* 2013, **13:**18.
41. Hulsmeier J, Pielage J, Rickert C, Technau GM, Klambt C, Stork T: **Distinct functions of alpha-Spectrin and beta-Spectrin during axonal pathfinding.** *Development* 2007, **134:**713-722.
42. Yang Z, Huh SU, Drennan JM, Kathuria H, Martinez JS, Tsuda H, Hall MC, Clemens JC: **Drosophila Vap-33 is required for axonal localization of Dscam isoforms.** *J Neurosci* 2012, **32:**17241-17250.
43. Rosenbaum EE, Hardie RC, Colley NJ: **Calnexin is essential for rhodopsin maturation, Ca2+ regulation, and photoreceptor cell survival.** *Neuron* 2006, **49:**229-241.
44. Yano T, Lopez de Quinto S, Matsui Y, Shevchenko A, Shevchenko A, Ephrussi A: **Hrp48, a Drosophila hnRNPA/B homolog, binds and regulates translation of oskar mRNA.** *Developmental Cell* 2004, **6:**637-648.
45. Berger J, Senti KA, Senti G, Newsome TP, Asling B, Dickson BJ, Suzuki T: **Systematic identification of genes that regulate neuronal wiring in the Drosophila visual system.** *PLoS Genet* 2008, **4:**e1000085.
46. Nelson MR, Luo H, Vari HK, Cox BJ, Simmonds AJ, Krause HM, Lipshitz HD, Smibert CA: **A multiprotein complex that mediates translational enhancement in drosophila.** *Journal of Biological Chemistry* 2007, **282:**34031-34038.
47. Goodrich JS, Clouse KN, Schupbach T: **Hrb27C, Sqd and Otu cooperatively regulate gurken RNA localization and mediate nurse cell chromosome dispersion in Drosophila oogenesis.** *Development* 2004, **131:**1949-1958.
48. Olesnicky EC, Killian DJ, Garcia E, Morton MC, Rathjen AR, Sola IE, Gavis ER: **Extensive Use of RNA-Binding Proteins in Drosophila Sensory Neuron Dendrite Morphogenesis.** *G3-Genes Genomes Genetics* 2014, **4:**297-306.
49. Olesnicky EC, Killian DJ, Garcia E, Morton MC, Rathjen AR, Sola IE, Gavis ER: **Extensive use of RNA-binding proteins in Drosophila sensory neuron dendrite morphogenesis.** *G3 (Bethesda)* 2014, **4:**297-306.
50. Buster DW, Nye J, Klebba JE, Rogers GC: **Preparation of Drosophila S2 cells for Light Microscopy.** 2010:e1982.
51. Adolph SK, DeLotto R, Nielsen FC, Christiansen J: **Embryonic expression of Drosophila IMP in the developing CNS and PNS.** *Gene Expression Patterns* 2009, **9:**138-143.
52. Kielpinski L, Boyd M, Sandelin A, Vinther J: **Detection of Reverse Transcriptase Termination Sites Using cDNA Ligation and Massive Parallel Sequencing.** In *Deep Sequencing Data Analysis. Volume* 1038. Edited by Shomron N: Humana Press; 2013: 213-231: *Methods in Molecular Biology*].
53. Konig J, Zarnack K, Rot G, Curk T, Kayikci M, Zupan B, Turner DJ, Luscombe NM, Ule J: **iCLIP--transcriptome-wide mapping of protein-RNA interactions with individual nucleotide resolution.** *J Vis Exp* 2011.
54. Hafner M, Landthaler M, Burger L, Khorshid M, Hausser J, Berninger P, Rothballer A, Ascano M, Jungkamp AC, Munschauer M, et al: **PAR-CliP--a method to identify transcriptome-wide the binding sites of RNA binding proteins.** *J Vis Exp* 2010.
55. Konig J, Zarnack K, Rot G, Curk T, Kayikci M, Zupan B, Turner DJ, Luscombe NM, Ule J: **iCLIP reveals the function of hnRNP particles in splicing at individual nucleotide resolution.** *Nat Struct Mol Biol* 2010, **17:**909-915.
56. Konig J, Zarnack K, Luscombe NM, Ule J: **Protein-RNA interactions: new genomic technologies and perspectives.** *Nat Rev Genet* 2011, **13:**77-83.

57. Flicek P, Amode MR, Barrell D, Beal K, Billis K, Brent S, Carvalho-Silva D, Clapham P, Coates G, Fitzgerald S, et al: **Ensembl 2014.** *Nucleic Acids Res* 2014, **42:**D749-755.
58. Kerpedjiev P, Frellsen J, Lindgreen S, Krogh A: **Adaptable probabilistic mapping of short reads using position specific scoring matrices.** *BMC Bioinformatics* 2014, **15:**100.
59. Baltz AG, Munschauer M, Schwanhausser B, Vasile A, Murakawa Y, Schueler M, Youngs N, Penfold-Brown D, Drew K, Milek M, et al: **The mRNA-bound proteome and its global occupancy profile on protein-coding transcripts.** *Mol Cell* 2012, **46:**674-690.
60. Kishore S, Jaskiewicz L, Burger L, Hausser J, Khorshid M, Zavolan M: **A quantitative analysis of CLIP methods for identifying binding sites of RNA-binding proteins.** *Nat Methods* 2011, **8:**559-564.
61. Anders S, Huber W: **Differential expression analysis for sequence count data.** *Genome Biol* 2010, **11:**R106.
